# History of Previous Midlife Estradiol Treatment Permanently Alters Interactions of Brain Insulin-like Growth Factor-1 Signaling and Hippocampal Estrogen Synthesis to Enhance Cognitive Aging in a Rat Model of Menopause

**DOI:** 10.1523/JNEUROSCI.0588-22.2022

**Published:** 2022-10-19

**Authors:** Nina E. Baumgartner, Shannon M. McQuillen, Samantha F. Perry, Sangtawan Miller, Matthieu J. Maroteaux, Robert B. Gibbs, Jill M. Daniel

**Affiliations:** ^1^Brain Institute; ^2^Neuroscience Program; ^3^Department of Psychology, Tulane University, New Orleans, Louisiana 70118; ^4^Department of Pharmaceutical Sciences, University of Pittsburgh School of Pharmacy, Pittsburgh, Pennsylvania 15261

**Keywords:** aging, estrogen, hippocampus, IGF-1, memory, menopause

## Abstract

Across species, including humans, elevated levels of brain estrogen receptor (ER) α are associated with enhanced cognitive aging, even in the absence of circulating estrogens. In rodents, short-term estrogen treatment, such as that commonly used in the menopausal transition, results in long-term increases in ERα levels in the hippocampus, leading to enhanced memory long after termination of estrogen treatment. However, mechanisms by which increased levels of brain ERα enhances cognitive aging remain unclear. Here we demonstrate in aging female rats that insulin-like growth factor-1 (IGF-1), which can activate ER via ligand-independent mechanisms, requires concomitant synthesis of brain-derived neuroestrogens to phosphorylate ERα via MAPK signaling, ultimately resulting in enhanced memory. In a rat model of menopause involving long-term ovarian hormone deprivation, hippocampal neuroestrogen activity decreases, altering IGF-1 activity and resulting in impaired memory. However, this process is reversed by short-term estradiol treatment. Forty days of estradiol exposure following ovariectomy results in maintenance of neuroestrogen levels that persist beyond the period of hormone treatment, allowing for continued interactions between IGF-1 and neuroestrogen signaling, elevated levels of hippocampal ERα, and ultimately enhanced memory. Collectively, results demonstrate that short-term estradiol use following loss of ovarian function has long-lasting effects on hippocampal function and memory by dynamically regulating cellular mechanisms that promote activity of ERα in the absence of circulating estrogens. Translational impacts of these findings suggest lasting cognitive benefits of short-term estrogen use near menopause and highlight the importance of hippocampal ERα, independent from the role of circulating estrogens, in regulating memory in aging females.

**SIGNIFICANCE STATEMENT** Declines in ovarian hormones following menopause coincide with increased risk of cognitive decline. Because of potential health risks, current recommendations are that menopausal estrogen therapy be limited to a few years. Long-term consequences for the brain and memory of this short-term midlife estrogen therapy are unclear. Here, in a rodent model of menopause, we determined mechanisms by which short-term midlife estrogen exposure can enhance hippocampal function and memory with cognitive benefits and molecular changes enduring long after termination of estrogen exposure. Our model indicates long-lasting benefits of maintaining hippocampal estrogen receptor function in the absence of ongoing estrogen exposure and suggests potential strategies for combating age-related cognitive decline.

## Introduction

Loss of ovarian hormones during menopause coincides with cognitive decline and increased risk of age-related dementias (Sherwin, 1994; Henderson et al., 1996). Because of putative health risks associated with prolonged estrogen exposure, current health guidelines recommend using menopausal estrogen treatment for the shortest time possible. Work from our laboratory in a rodent model of menopause has demonstrated long-lasting benefits of short-term midlife estradiol treatment on hippocampal function and memory through sustained activation of estrogen receptor (ER) α that are likely permanent, persisting long after estradiol treatment is terminated (Rodgers et al., 2010; Witty et al., 2013; Black et al., 2016; Baumgartner et al., 2021). These findings correspond with evidence across multiple species, including humans, that elevated levels of brain estrogen receptor ERα are associated with enhanced cognitive aging even in the absence of circulating estrogens (for review, see Baumgartner and Daniel, 2021). The mechanisms by which increased levels of brain ERα enhance cognitive aging following previous midlife exposure to estradiol are unclear.

Short-term exposure to estradiol in midlife enhances memory and increases levels of hippocampal ERα long-term in ovariectomized rodents (Rodgers et al., 2010), effects shown to be dependent on insulin-like growth factor-1 (IGF-1) signaling (Witty et al., 2013), resulting in sustained ER-dependent transcriptional activity (Pollard et al., 2018). IGF-1 is a peptide hormone that acts via IGF-1R, a tyrosine kinase receptor with much functional overlap with ERα, including activation of MAPK and PI3K-AKT signaling pathways by both receptors (Russo et al., 2005; Sohrabji, 2015). ERα and IGF-1R colocalize and form estradiol-dependent protein complexes in the hippocampus (Cardona-Gómez et al., 2000; Mendez et al., 2003). Implications for these subcellular interactions for cognition remain to be determined. IGF-1 administration activates ERα via ligand-independent mechanisms *in vitro* (Kato et al., 1995) and in recently ovariectomized rats via phosphorylation at serine-118 (Grissom and Daniel, 2016), a phospho-site crucial for protecting ERα from degradation (Valley et al., 2005). An *in vitro* study revealed that neuroestrogen synthesis is required for IGF-1-mediated activation of ERα, potentially through synergistic activation of the MAPK pathway (Pollard and Daniel, 2019).

Contradicting a potential role for neuroestrogens in activation of ERα following loss of ovarian function are data indicating that hippocampal neuroestrogens are regulated by circulating estrogens (Nelson et al., 2016) and demonstrations of decreases in hippocampal aromatase expression and neuroestrogen levels following long-term ovariectomy (Ma et al., 2020; Chen et al., 2021). Additionally, we showed that blocking neuroestrogen synthesis via aromatase inhibition had no impact on hippocampal ER-dependent transcription in long-term ovariectomized mice (Baumgartner et al., 2019). Collectively, these data point to a diminished role for neuroestrogen synthesis in hippocampal function following long-term ovarian hormone deprivation.

In summary, previous research demonstrates that a history of midlife estradiol treatment impacts memory long after termination of estradiol treatment through lasting activation of hippocampal ERα by ligand-independent mechanisms via IGF1 signaling. *In vitro* evidence indicates that ligand-independent activation of ER by IGF-1 requires concomitant neuroestrogen synthesis. However, neuroestrogen levels in the hippocampus may be diminished following long-term loss of ovarian hormones. The goal of the current work was to reconcile these contradictory findings and determine implications for female cognitive aging following loss of ovarian function of the interactive actions of IGF-1 and neuroestrogens and determine whether history of estradiol use impacts that interaction. First, we determined the necessity of neuroestrogen synthesis in the ability of IGF-1 to activate ERα *in vivo* via its downstream signaling pathways and subsequent impact on memory. Next, we determined whether interactions of neuroestrogens and IGF-1 in the hippocampus and subsequent impact on memory were altered in two models of menopause: one with and one without a history of past midlife estradiol use. Our findings provide a potential model for combatting postmenopausal cognitive decline in which short-term estradiol treatment following the loss of ovarian hormones sustains hippocampal function and memory well beyond the period of estradiol exposure by permanently altering the dynamic relationship between IGF-1R signaling and neuroestrogen synthesis.

## Materials and Methods

### Subjects

Middle-aged female Long-Evans hooded rats (Envigo), retired breeders (∼11 months of age), were used for all experiments. Animal care was in accordance with guidelines set by the National Institutes of Health's *Guide for the care and use of laboratory animals* (National Institutes of Health, 2011), and all procedures were approved by the Institutional Animal Care and Use Committee of Tulane University. Rats were housed individually in a temperature-controlled vivarium under a 12 h light, 12 h dark cycle and had unrestricted access to food and water unless otherwise noted. All experimental procedures occurred during their light cycle.

### Ovariectomies and hormone treatments

All rats in experiments were anesthetized by intraperitoneal injections of ketamine (100 mg/kg i.p.; Bristol Laboratories) and xylazine (7 mg/kg i.p.; Miles Laboratories) and ovariectomized. Buprenorphine (0.375 mg/kg; Reckitt Benckiser Health Care) was administered by subcutaneous injection before the start of each surgery. Ovariectomy surgery involved bilateral flank incisions through the skin and muscle wall and the removal of ovaries.

For Experiments 3 and 4, rats were implanted with a subcutaneous 5 mm Silastic brand capsule (0.058 inch inner diameter and 0.077 inch outer diameter; Dow Corning) on the dorsal aspect of the neck immediately following ovariectomy. Capsules contained either cholesterol vehicle (Experiment 3; Sigma-Aldrich) or 25% 17β-estradiol (Experiment 4; Sigma-Aldrich) diluted in vehicle. We have previously shown that implants of these dimensions and estradiol concentrations maintain blood serum estradiol levels in middle-age retired breeders at ∼37 pg/ml (Bohacek and Daniel, 2007), which falls within physiological range. Forty days after ovariectomy and capsule implantation, capsules were removed. Vaginal smears for each rat were collected for at least 4 consecutive days before capsule replacement to confirm hormone treatment for the initial 40 d window. Smears of ovariectomized, cholesterol-treated rats were characterized by a predominance of leukocytes, while smears of ovariectomized, estradiol-treated rats were characterized by a predominance of cornified and nucleated epithelial cells.

### Stereotaxic surgeries

Rats were anesthetized with ketamine and xylazine as described above and administered buprenorphine as an analgesic. Rats were then placed into a stereotaxic frame. An incision was made in the scalp and fascia that overlie the skull, and a hole was drilled in the skull.

In Experiment 1, a cannula connected to a Hamilton syringe via Silastic tubing was lowered through the hole to the appropriate depth to reach the right lateral ventricle (relative to bregma: anteroposterior, −0.5 mm; mediolateral, −1.1 mm; dorsoventral, −2.5 mm). Cannulas delivered 5 µl of either vehicle containing 8% DMSO (Sigma-Aldrich) in aCSF (Tocris), 2 µg of human IGF-1 (GroPep) diluted in vehicle, or 2 µg of IGF-1 combined with 0.4 µg of aromatase inhibitor letrozole (Sigma-Aldrich) diluted in vehicle over the course of 10 min. Following infusions, cannulas were slowly raised out of skull and wounds were sutured closed.

In Experiments 2-4, a cannula (brain infusion kits, Alzet #0008663) was lowered through the hole to the appropriate depth to reach the right lateral ventricle (relative to bregma: anteroposterior, −0.3 mm; mediolateral, −1.2 mm; dorsoventral, −4.5 mm) and adhered to the skull with an anchoring screw, Super Glue, and dental acrylic. The cannula was connected to an osmotic mini-pump (flow rate 0.15 µl/h, max volume 200 µl; Alzet #2006) by vinyl tubing for drug delivery. Rats in Experiment 2 received mini-pumps that delivered vehicle containing 6.7% DMSO in aCSF, human IGF-1 (0.33 µg/µl) diluted in vehicle, or human IGF-1 (0.33 µg/µl) and letrozole (0.066 µg/µl) diluted in vehicle. Rats from Experiments 3 and 4 received mini-pumps that delivered vehicle (8% DMSO in aCSF), IGF-1 receptor antagonist JB1 in vehicle (300 µg/ml, Bachem), aromatase inhibitor letrozole in vehicle (0.066 µg/µl), or both JB1 + letrozole in vehicle.

### Radial-arm maze training

Approximately 1 week before the start of behavioral training, rats were food-restricted and weighed daily to maintain their body weights at 85%-90% of their free-feeding weight. Rats then began training on the 8-arm radial-maze task (Coulbourn Instruments), as previously described (Daniel, 2015). The maze consists of eight arms (66 cm long × 9.5 cm wide × 11.5 cm high) with a metal grated floor and clear acrylic walls. Arms extend out radially from a central hub that is 28 cm in diameter, and the maze was placed on a table that is ∼1 m above the ground. The maze was centered in a 3 × 5 m room with many visible extra maze cues. During training, a single food reward (Froot Loops; Kellogg) was placed in an opaque dish, 5.5 cm in diameter and 1.25 cm tall, at the end of each arm so it was not visible from the center of the maze. For each trial, the rat was placed in the center of the maze facing one of the eight arms. The starting orientation varied pseudo-randomly across trials. The rat was then allowed to enter arms and obtain food rewards until all eight arms had been visited or 5 min had elapsed. An arm entry was scored when all four paws crossed the midline of the arm. The arm entry sequence was scored in real time by an observer located in a fixed location in the room. Errors were scored if the rat re-entered an arm that had already been visited previously in the trial. Rats were trained with one trial per day, 5 d per week, for up to 25 d until they reached criterion by scoring <2 errors for 3 consecutive days. Once criterion was reached, rats underwent stereotaxic surgery and drug delivery as described above.

### Delay testing on radial-arm maze

One week after stereotaxic surgeries, rats were tested on delay trials. During testing, delays of various lengths were imposed, which required the rats to remember over an extended period of time which arms had previously been visited. Rats were placed in the center of the maze facing one of the eight arms and allowed to enter four unique arms during the predelay trial. After four correct arm choices, rats were removed from the maze and placed in a holding cage for the duration of the delay. Following the delay, rats were returned to the center of the maze in the same orientation from the predelay trial. During this postdelay trial, rats were allowed to explore the maze until the four remaining, still baited arms were visited or until 5 min had elapsed. Re-entries into previously visited arms were recorded as errors. Arm-choice accuracy was measured by errors of 8, which represented the number of errors included in the first eight arm choices collectively across the predelay and postdelay trials. Rats received 2 d of habituation to a 1 min delay trial. Each subsequent delay was tested across 2 consecutive days. Delays for each experiment were chosen based on the performance of the rats during the training period and were increased in difficulty until at least two experimental groups performed within 1 SD from chance (2.7 errors of 8). Means of errors of 8 across both days of testing for each delay were analyzed.

### Death and tissue collection

Rats were killed under anesthesia induced by ketamine and xylazine. The hippocampus was dissected out and quick frozen on dry ice and stored at −80°C until further processing. A 1 cm sample of the right uterine horn was collected from each rat and weighed to verify ovariectomy status or hormone treatment at the time of death.

### Tissue processing and Western blotting

In Experiment 1, right hippocampi were processed for subcellular protein fractionation and compartment-specific Western blotting as previously described (Baumgartner et al., 2021). Briefly, hippocampal tissue was homogenized using the PowerGen-125 handheld homogenizer (Fisher), strained through Pierce Tissue Strainers, and separated into cytosolic, membrane, and nuclear compartments using consecutive centrifugation steps of varying speeds and specialized buffers obtained from a commercially available kit (Sub-Cellular Protein Fractionation Kit for Tissues, Thermo Fisher Scientific). Bradford protein assays were performed for each compartment individually. Each sample was diluted 1:1 with Laemmli Sample Buffer (Bio-Rad) mixed with 350 mm DTT, boiled for 5 min, and stored at −80°C until Western blotting.

The left hippocampi in Experiment 1 and the right hippocampi in Experiments 2-4 were processed for whole-cell Western blotting. Tissue was sonicated using the Fisherbrand Model 50 Sonic Dismembrator (Fisher) in 10 µl/mg lysis buffer containing 1 mm EGTA, 1 mm EDTA, 20 mm Tris, 1 mm sodium pyrophosphate tetrabasic decahydrate, 4 mm 4-nitrophenyl phosphate disodium salt hexahydrate, 0.1 µm microcystin, and 1% protease inhibitor cocktail (Sigma-Aldrich). Samples were then centrifuged for 15 min at 1000 × *g* at 4°C. Bradford protein assays were performed to determine the protein concentration of each sample. Each sample was diluted 1:1 with Laemmli Sample Buffer (Bio-Rad) mixed with 350 mm DTT, boiled for 10 min, and stored at −80°C until Western blotting.

Fifteen micrograms of cytosolic, membrane, and nuclear protein from each sample, or 25 µg of whole-cell protein from each sample, was loaded onto and separated on a 7.5% TGX SDS-PAGE gel at 250 V for 40 min. Molecular weight markers (Precision Plus Protein Standards, Bio-Rad) were included with each run. Proteins were transferred from gels to nitrocellulose membranes at 100 V for 30 min. Membranes were blocked with 5% BSA in 1% Tween 20/TBS (TTBS) with gentle mixing at room temperature for 1 h. After blocking, membranes were incubated with gentle mixing in primary antibody overnight at 4°C in 1% BSA-TTBS. Following primary antibody incubation, blots were washed 3 times for 15 min with TTBS. Blots were then incubated with secondary antibodies conjugated to fluorophores in 5% BSA-TTBS for 1 h at room temperature with gentle mixing. Blots were washed 3 times for 15 min with TTBS, and then imaged on the ChemiDocMP set to channels for StarBright B520 and StarBright B700 using the auto-optimization function. MCID Core imaging software was used to quantify optical density × area (D×A) for bands of interest, using the MCID autoscan A function in which background optical density is automatically detected and subtracted from band intensity and multiplies the relative intensity by the area of the band.

### Antibodies

Samples from cytosolic, membrane, and nuclear compartments were incubated with primary antibodies for phospho-S118 ERα (1:1000; Abcam, rabbit monoclonal, ab32396; ∼66 kDa) (Moriel-Carretero et al., 2011) and total ERα (1:1000, Santa Cruz Biotechnology, mouse monoclonal, sc-71064; ∼66 kDa) (Liu et al., 2017). Samples from cytosolic fractions were incubated with antibodies for cytosolic loading control enolase (1:1000, Santa Cruz Biotechnology, mouse monoclonal, sc-271384; ∼48 kDa). Samples from membrane fractions were incubated with antibodies for membrane loading control ATP1A1 (1:5000, ProteinTech, rabbit polyclonal, 14418-1-AP, ∼110 kDa). Samples from nuclear fractions were incubated with antibodies for nuclear loading control histone 3 (H3; 1:1000, Cell Signaling Technology, rabbit polyclonal #9715, ∼17 kDa). Whole-cell tissue samples were incubated with antibodies for phospho-MAPK (1:1000, Cell Signaling Technology, mouse monoclonal #4696, ∼44 and ∼42 kDa bands) (Nabet et al., 2020) and total MAPK (1:1000, Cell Signaling Technology, rabbit monoclonal #4370, ∼44 and ∼42 kDa bands) (Nabet et al., 2020), which recognize both the p44- and p42-MAPK epitopes, phospho-Akt (1:1000, Cell Signaling Technology, rabbit monoclonal #4060,∼60 kDa) (Nabet et al., 2020), total Akt (1:1000, Cell Signaling Technology, mouse monoclonal, #2920, ∼60 kDa) (Nabet et al., 2020), total ERα (1:1000, Santa Cruz Biotechnology, mouse monoclonal, sc-71064; ∼66 kDa) (Liu et al., 2017), aromatase (1:1000, Bio-Rad, mouse monoclonal, MCA2077S, ∼55 kDa) (Inaoka et al., 2008), or loading control GAPDH (1:1000, Santa Cruz Biotechnology, mouse monoclonal, sc-32233, ∼37 kDa).

Primary antibodies used to detect changes in signaling pathways (MAPK, p-MAPK, AKT, p-AKT) have been used extensively by us (Witty et al., 2013; Pollard and Daniel, 2019) and others (Nabet et al., 2020) to demonstrate sensitivity of these pathways to pharmacological and hormonal manipulations. Primary antibodies used to detect ERα and pS118-ERα have also been used by us (Grissom and Daniel, 2016; Baumgartner et al., 2021) and others (Moriel-Carretero et al., 2011; Liu et al., 2017) to reliably demonstrate the impact of estradiol and IGF-1 manipulations on their levels. Furthermore, we tested the specificity of our ERα antibody in brain tissue from ERα KO mice that revealed a lack of the expected ∼66 kDa band compared with tissue from WT mice and rats. We have not previously published with the antibody used to detect aromatase used here. However, it has been reliably used by others (Ghosh et al., 2008; Inaoka et al., 2008) to demonstrate sensitivity of aromatase levels to intracellular signaling pathways and genetic manipulations. Additionally, we regularly validate all antibodies in our laboratory using primary-only and secondary-only control blots.

Secondary antibodies used were StarBright B520 Rabbit (Bio-Rad, #12005869; 1:5000 for p-S118 ERα, enolase, ATP1A1, H3, total MAPK, total Akt) and StarBright B700 Mouse (Bio-Rad, #12004158; 1:5000 for total ERα, phospho-MAPK, phospho-Akt, aromatase, GAPDH).

### Hippocampal estradiol detection

Left hippocampi from Experiments 3 and 4 were processed for estradiol extraction and measurement via UPLC-MS/MS as previously described and validated (Li et al., 2016). This method has recently been shown to sensitively detect estradiol levels in hippocampal tissue from ovariectomized rats treated with estradiol in a dose-dependent manner (Li and Gibbs, 2019). Briefly, tissues were homogenized in a potassium PB (0.12 m, pH 7.4; 100 mg tissue/ml) containing 4.0 mm MgCl_2_, 4.0 mm Tris, and 50 mm sucrose. Samples were spiked with deuterated 17β-estradiol and then extracted with n-butyl chloride (Sigma-Aldrich). The organic layer was dried under nitrogen, then resuspended and derivatized with dansyl chloride in a 1:1 mix of acetonitrile:water (pH 10.5, Sigma-Aldrich). Samples were then centrifuged and the supernatant transferred to glass vials for UPLC-MS/MS analysis. Calibration curves were prepared in a matrix of 0.2% 2-hydroxypropyl-ß-cyclodextrin and processed the same as the tissue extracts.

Estradiol was eluted using a Waters Acquity UPLC BEH C18, 1.7 µm, 21 × 150 mm reversed-phase column, with an acetonitrile:water (0.1% formic acid) gradient. Detection was in the positive mode. Transitions used for analysis were 506→171 for estradiol, and 511→171 for the internal standard. This method is able to distinguish between 17α- and 17β-estradiol based on retention time. Limit of detectability is 0.009 pmol/ml (2.5 pg/ml) with intraday and interday relative SDs of <15% at all concentrations.

### Experimental design and statistical analyses

#### Experiment 1

**Figure 1.**
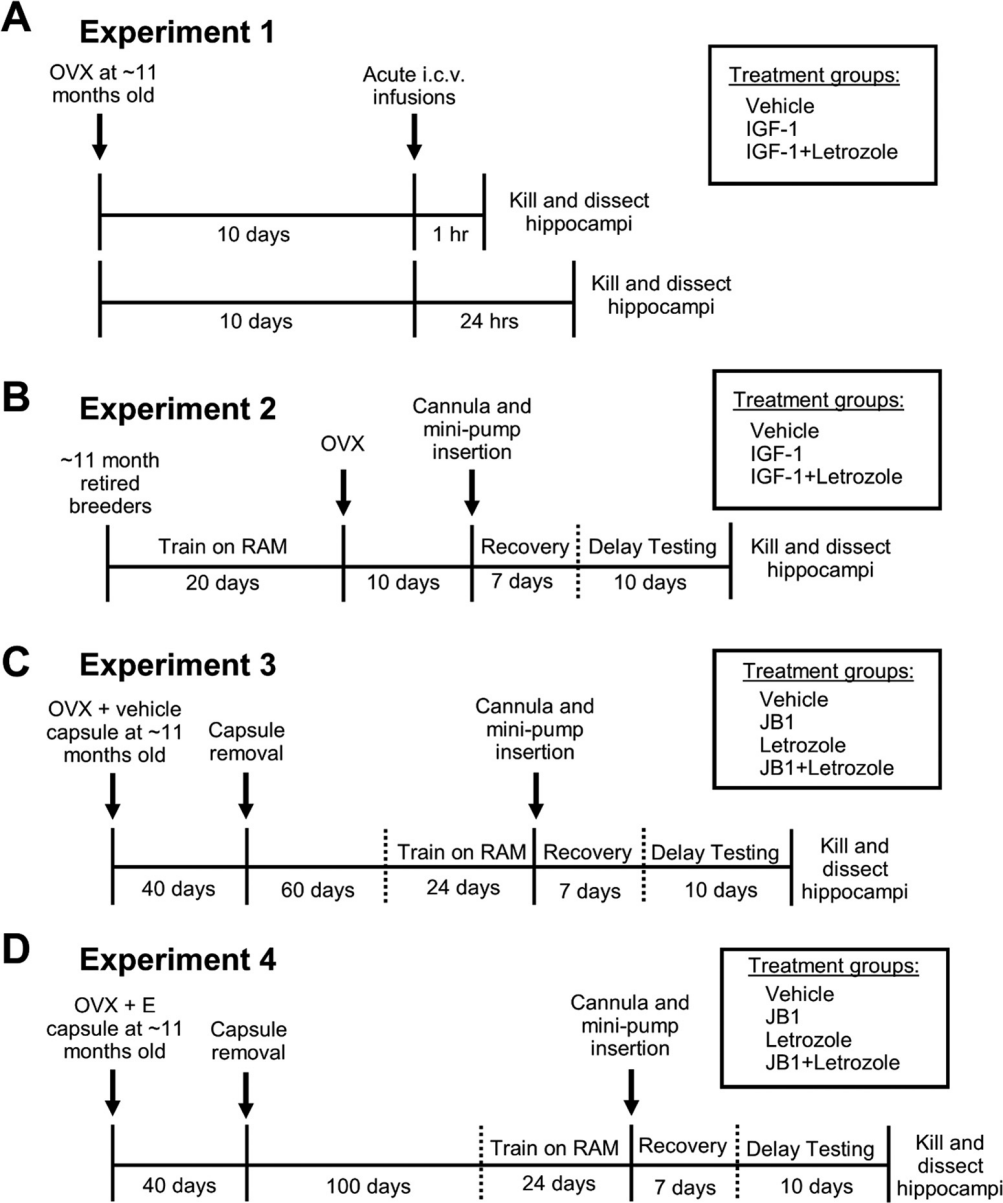
Experimental timelines. ***A***, In Experiment 1, middle-aged female rats were ovariectomized (OVX) and 10 d later received acute intracerebroventricular (i.c.v.) infusions of vehicle, IGF-1, or IGF-1 plus aromatase inhibitor letrozole. Either 1 or 24 h later, rats were killed and hippocampi were processed for Western blotting. ***B***, In Experiment 2, middle-aged female rats were trained on the radial-arm maze (RAM) for 20 d before undergoing ovariectomy. Ten days later, rats were implanted with cannulas directed to the lateral ventricle connected to osmotic mini-pumps delivering chronic administration of vehicle, IGF-1, or IGF-1 plus letrozole. Following 1 week of recovery, rats were tested on the RAM with delays of increasing difficulty. After completion of delay testing, rats were killed and hippocampi were processed for Western blotting. ***C***, In Experiment 3, middle-aged female rats were ovariectomized and implanted with subcutaneous vehicle capsules. Forty days later, capsules were removed. Sixty days after capsule removal, rats were trained on the RAM for 24 d. Then, rats were implanted with cannulas directed to the lateral ventricle connected to osmotic mini-pumps delivering chronic administration of vehicle, IGF-1R antagonist JB1, letrozole, or JB1+Let. Following 1 week of recovery, rats were tested on the RAM with delays of increasing difficulty. After completion of delay testing, rats were killed and hippocampi were processed for Western blotting and estradiol level detection. ***D***, In Experiment 4, middle-aged female rats were ovariectomized and implanted with subcutaneous estradiol capsules. Forty days later, capsules were removed. One hundred days after capsule removal, rats were trained on the RAM for 24 d. Then, rats were implanted with cannulas directed to the lateral ventricle connected to osmotic mini-pumps delivering chronic administration of either vehicle, IGF-1R antagonist JB1, letrozole, or JB1+Let. Following 1 week of recovery, rats were tested on the RAM with delays of increasing difficulty. After completion of delay testing, rats were killed and hippocampi were processed for Western blotting and estradiol level detection.

As illustrated in Figure 1*A*, middle-aged rats were ovariectomized and 10 d later treated them with an acute intracerebroventricular infusion of vehicle, IGF-1, or IGF-1 plus letrozole, an aromatase inhibitor that blocks estrogen synthesis. After 1 h (Vehicle, *n* = 9; IGF-1, *n* = 9; IGF-1+Let, *n* = 10) or 24 h (Vehicle, *n* = 10; IGF-1, *n* = 9; IGF-1+Let, *n* = 9), animals were killed and hippocampi were dissected. Right hippocampal tissue was collected and processed for subcellular fractionation and Western blotting for phospho-S118 ERα and total ERα. Left hippocampal tissue was collected and processed for whole-cell Western blotting for phospho-p42-MAPK/total p42-MAPK and phospho-Akt/total Akt to determine whether blocking neuroestrogen synthesis decreases MAPK and PI3K-Akt signaling in animals simultaneously treated with IGF-1.

#### Experiment 2

As illustrated in Figure 1*B*, middle-aged rats were trained on the 8-arm radial maze for 21 d before undergoing ovariectomy. Ten days after ovariectomy, rats were implanted with a cannula and mini-pump, which chronically delivered vehicle (*n* = 11), IGF-1 (*n* = 9), or IGF-1 plus letrozole (*n* = 11) to the lateral ventricle for the duration of the experiment. Animals were then tested on delay trials (1 min, 1, 3, 4, 5 h) in the 8-arm radial maze to test hippocampal-dependent spatial memory. Two days after the final day of delay testing, animals were killed, and right hippocampal tissue was collected and processed for whole-cell Western blotting for phospho-p42-MAPK/total p42-MAPK and phospho-Akt/total to determine whether chronic letrozole treatment impacts hippocampal activation of the MAPK and PI3K-AkT pathways in animals treated with IGF-1.

#### Experiment 3

As illustrated in Figure 1*C*, middle-aged rats were ovariectomized and immediately implanted with subcutaneous vehicle capsules for 40 d (to match subsequent estradiol treatments in Experiment 4). Forty days later, capsules were removed. Animals were allowed to age for 60 more days following capsule removal before behavioral training begin, resulting in a total of 100 d between removal of estrogens (ovariectomy) and behavioral training. Following that 60 d waiting period, animals were trained on the radial-arm maze for 24 d. Animals then underwent stereotaxic surgery and were implanted with a cannula and mini-pump that chronically delivered vehicle (*n* = 10), IGF-1 receptor antagonist JB1 (*n* = 10), aromatase inhibitor letrozole (*n* = 9), or JB1 and letrozole (*n* = 9). Animals were tested on delay trials (No delay, 1 min, 1 h) in the radial-arm maze to test hippocampal-dependent spatial memory. Two days after the final day of delay testing, animals were killed and right hippocampal tissue was collected and processed for Western blotting for ERα, aromatase, phospho-p42-MAPK, total p42-MAPK, phospho-Akt, and total-Akt. Left hippocampal tissues were collected and processed for estradiol detection via UPLC-MS/MS.

#### Experiment 4

As illustrated in Figure 1*D*, middle-aged rats were ovariectomized and immediately implanted with subcutaneous estradiol capsules. Forty days later, capsules were removed. Although we have previously demonstrated that the behavioral effects of this short-term estradiol exposure are unique from simply remaining ovary intact for the same amount of time (Black et al., 2018), we still sought to control for the overall time between the loss of circulating estrogens and beginning of behavioral testing across experiments. Therefore, to match the 100 d postovariectomy period in Experiment 3, animals in Experiment 4 were allowed to age for 100 d following capsule removal before behavioral training began, resulting in a total of 100 d between removal of estrogens (capsule removal) and behavioral training. Following the 100 d waiting period, animals were trained on the radial-arm maze for 24 d. Animals then underwent stereotaxic surgery and were implanted with a cannula and mini-pump that chronically delivered vehicle (*n* = 9), IGF-1 receptor antagonist JB1 (*n* = 8), aromatase inhibitor letrozole (*n* = 9), or JB1 and letrozole (*n* = 9). Animals were tested on delay trials (No delay, 1 min, 1, 2, and 3 h) in the 8-arm radial maze to test hippocampal-dependent spatial memory. Two days after the final day of delay testing, animals were killed and right hippocampal tissue was collected and processed for Western blotting for ERα, aromatase, phospho-p42-MAPK, total p42-MAPK, phospho-Akt, and total-Akt were performed. Left hippocampal tissue were collected and processed for estradiol detection via UPLC-MS/MS.

### Statistical analyses

All data analyses were performed using SPSS software. Behavioral data were analyzed by mixed-design ANOVA comparing errors of 8 between treatment groups and across delay trials. Subsequent *post hoc* testing, as described below, was used as appropriate for between-subject effects. Western blotting and mass spec data were analyzed by one-way ANOVA comparing optical density and estradiol levels in fmol/ml, respectively, between experiment groups with subsequent *post hoc* testing as appropriate.

For experiments with only three experimental groups (Experiment 1 and 2), LSD *post hoc* testing was used as appropriate for between-group effects. For experiments with more than three experimental groups (Experiments 3 and 4), a significant main effect of treatment was probed by the Dunnett's two-sided *post hoc* test, which compares treatments with a single control group (Vehicle group). Western data were analyzed by one-way ANOVA comparing optical density between treatment group and subsequent *post hoc* testing as appropriate. For quantification of estradiol levels, two samples from the letrozole group in Experiment 3 were used for spike and recovery tests to optimize procedures for this set of samples and were therefore excluded from statistical analysis. Additionally, because of the high sensitivity of mass spec detection, extreme statistical outliers as identified by SPSS software (defined as ±3 × interquartile range from the first or third quartiles for each group) were presumed to indicate sample contamination and therefore were excluded from statistical analyses. Researchers were blind to treatment group during behavioral testing, Western blotting, mass spec, and data analysis.

## Results

### Experiment 1: role of neuroestrogens in the ability of IGF-1 to activate ERα

In the absence of ovarian estrogens, IGF-1 activates ERα via ligand-independent mechanisms (Kato et al., 1995; Grissom and Daniel, 2016). IGF-1 activation of ERα *in vitro* requires concomitant neuroestrogen synthesis (Pollard and Daniel, 2019). The goal of this experiment was to test the hypothesis that neuroestrogen synthesis is required for IGF-1 activation of ERα protein *in vivo*. Recently ovariectomized middle-aged female rats received intracerebroventricular infusions of vehicle (Veh group), IGF-1 (IGF-1 group), or IGF-1 plus letrozole (IGF-1+Let group). Total and phosphorylated levels of ERα and the IGF-1 regulated signaling proteins MAPK and AKT were measured either 1 or 24 h after infusion.

#### Phosphorylation of ERα

As a nuclear steroid receptor with the ability to be inserted into cell membranes, the subcellular localization of ERα impacts the receptor's function. Therefore, we measured levels of phosphorylated and total ERα levels in the cytosol, membrane, and nuclear compartments of hippocampal cells at each time point.

**Figure 2.**
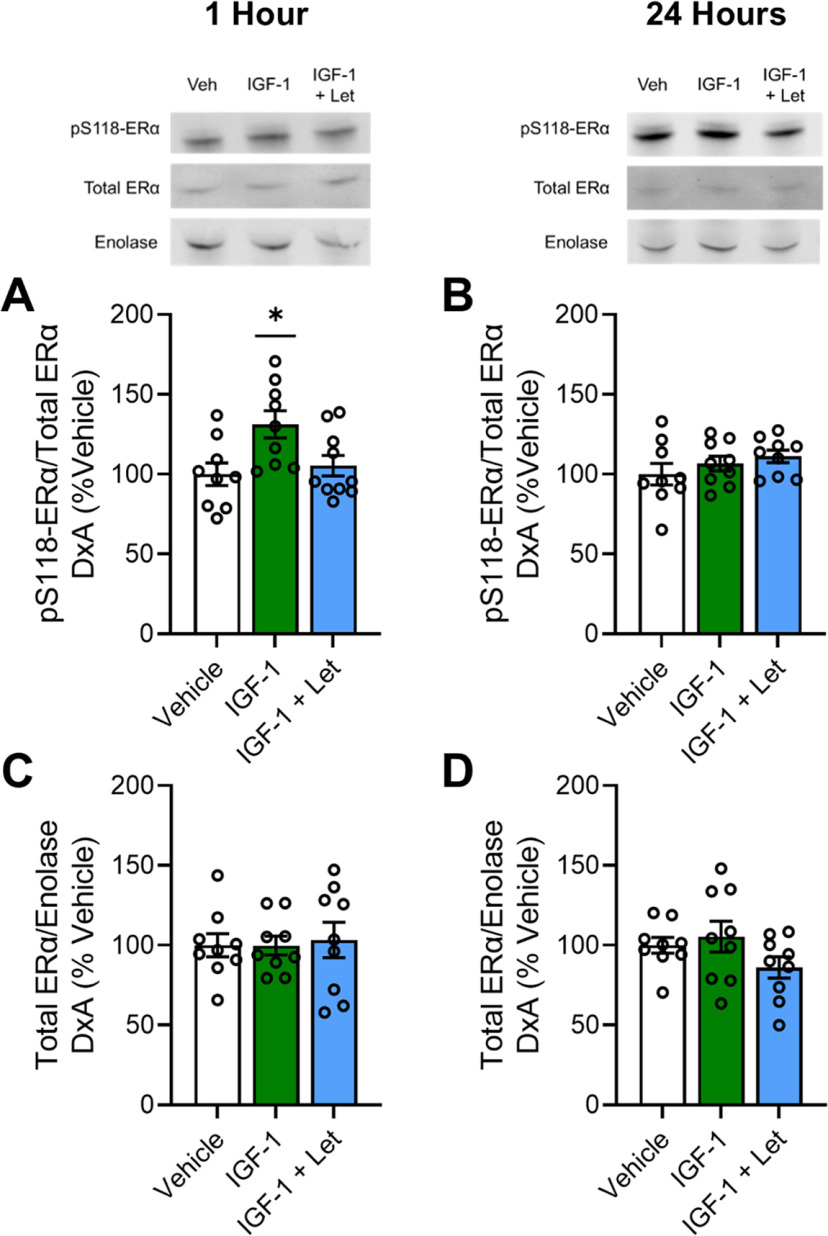
Cytosolic expression of pS118-ERα and total ERα 1 or 24 h after infusion of IGF-1 or IGF-1+Let in the hippocampus of ovariectomized rats. Middle-aged female rats were ovariectomized and given an acute infusion of Veh, IGF-1, or IGF-1+Let to the lateral ventricle. Either 1 or 24 h later, hippocampi were processed for subcellular fractionation and Western blotting for phosphorylated levels of pS118-ERα, total ERα, and cytosolic loading control enolase in the cytosolic fraction of all samples. Levels of pS118 were normalized to total ERα levels, and levels of total ERα were normalized to enolase levels. Graph represents mean D×A ± SEM expressed as a percentage of the vehicle group. ***A***, There was an effect of treatment (*p* < 0.05) on pS118-ERα levels in the cytosol compartment 1 h after infusion, with *post hoc* testing revealing increased levels in the IGF-1 group compared with vehicle group. ***B–D***, There was no effect of treatment on pS118-ERα levels 24 h after infusion (***B***), nor was there an effect of treatment on total ERα levels either 1 h (***C***) or 24 h (***D***) after infusion. *p < 0.05 versus Veh.

In the cytosolic compartment (Fig. 2), there was a main effect of treatment on levels of pS118-ERα (*F*_(2,27)_ = 4.973; *p* = 0.015) at 1 h after treatment (Fig. 2*A*), with levels of pS118-ERα significantly increased in the IGF-1 treatment group compared with the vehicle group (*p* = 0.007) and the IGF-1+Let treatment group (*p* = 0.020). This observed increase in phosphorylated ERα in the cytosol after 1 h is consistent with earlier work in cell cultures demonstrating that peak dimerization (and presumably, therefore, nuclear translocation) of ERα does not occur until 2 h after estrogen treatment (Powell et al., 2010). However, there was no significant difference in cytosolic pS118-ERα levels between the IGF-1+Let and vehicle groups (*p* = 0.611). There was no effect of treatment on total levels of cytosolic ERα 1 h after infusion (Fig. 2*B*; *F*_(2,27)_ = 0.553, *p* = 0.582). At the 24 h time point, there were no effects of treatment on cytosolic levels of pS118-ERα (Fig. 2*C*; *F*_(2,27)_ = 0.292, *p* = 0.750) or total ERα (Fig. 2*D*; *F*_(2,27)_ = 1.408, *p* = 0.263).

**Figure 3.**
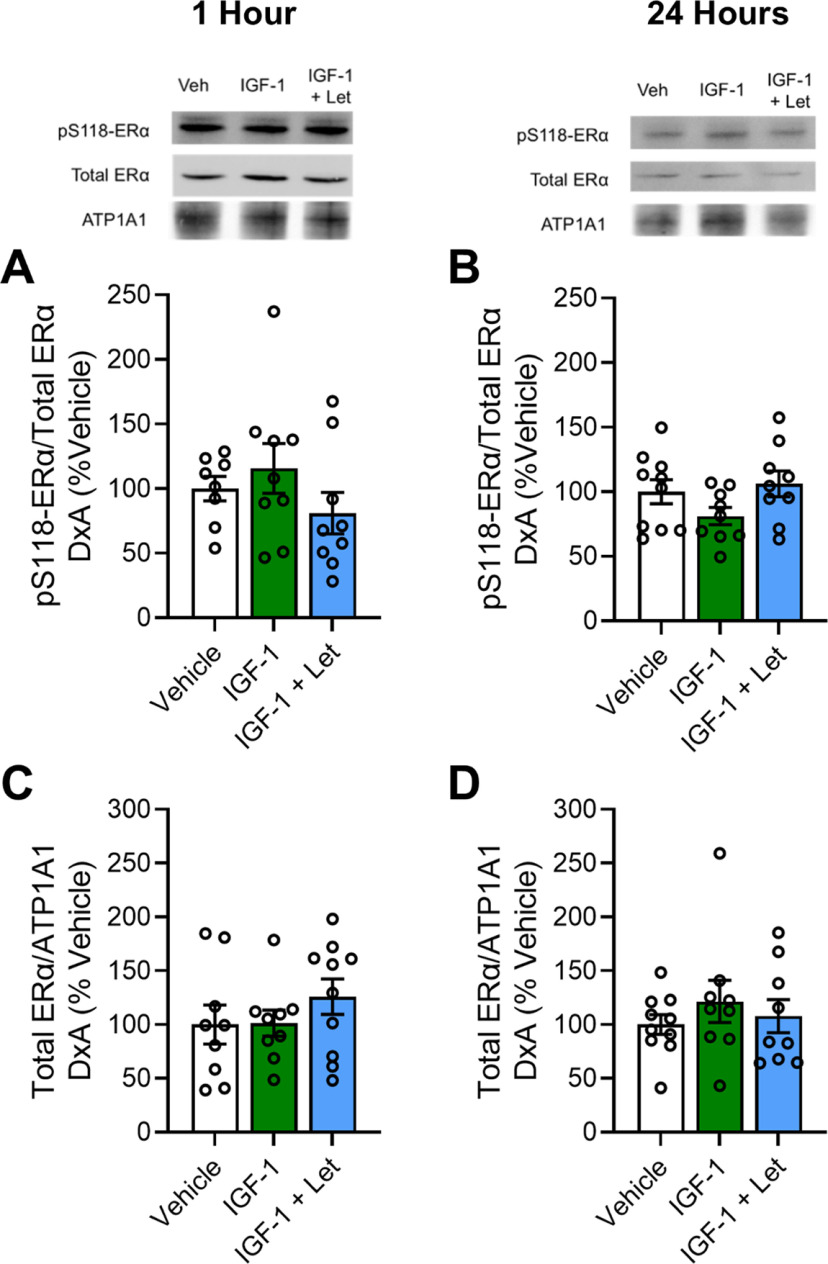
Membrane expression of pS118-ERα and total ERα 1 or 24 h after infusion of IGF-1 or IGF-1+Let in the hippocampus of ovariectomized rats. Middle-aged female rats were ovariectomized and given an acute infusion of Veh, IGF-1, or IGF-1 and IGF-1+Let to the lateral ventricle. Either 1 or 24 h later, hippocampi were processed for subcellular fractionation and Western blotting for phosphorylated levels of pS118-ERα, total ERα, and membrane loading control ATP1A1 in the membrane fraction of all samples. Levels of pS118-ERα were normalized to total ERα levels, and levels of total ERα were normalized to ATP1A1 levels. Graph represents mean D×A ± SEM expressed as a percentage of the vehicle group. ***A***, ***B***, There was no effect of treatment on membrane pS118-ERα levels either 1 h (***A***) or 24 h (***B***) after infusion. ***C***, ***D***, There was no effect of treatment on membrane total ERα levels either 1 h (***C***) or 24 h (***D***) after infusion.

In the membrane compartment (Fig. 3), there were no effects of treatment 1 h after infusion on levels of pS118-ERα (Fig. 3*A*, *F*_(2,25)_ = 1.243; *p* = 0.307) or total ERα (Fig. 3*B*, *F*_(2,27)_ = 0.875; *p* = 0.429). There were also no effects of treatment 24 h after infusion on membrane levels pS118-ERα (Fig. 3*C*, *F*_(2,27)_ = 2.122; *p* = 0.141) or total ERα (Fig. 3*D*, *F*_(2,27)_ = 0.528; *p* = 0.596).

**Figure 4.**
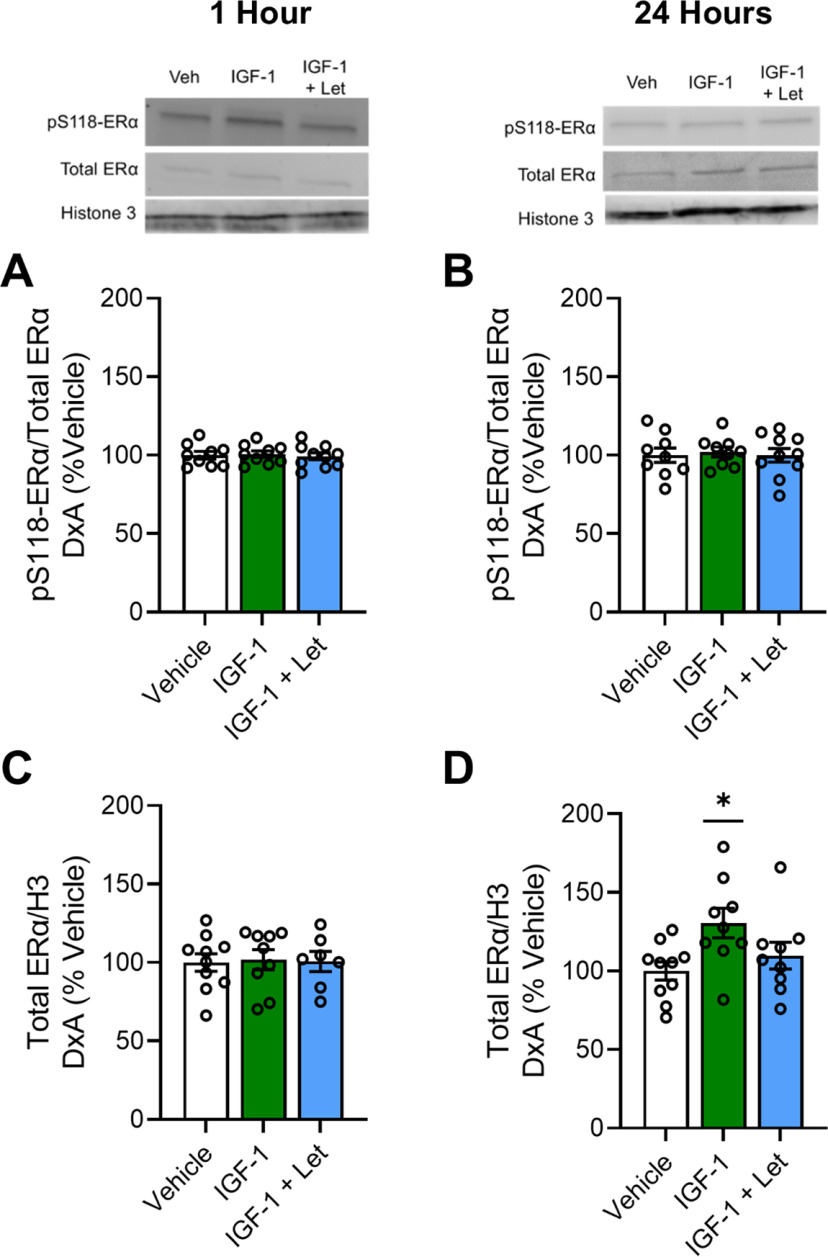
Nuclear expression of pS118-ERα and total ERα 1 or 24 h after infusion of IGF-1 or IGF-1+Let in the hippocampus of ovariectomized rats. Middle-aged female rats were ovariectomized and given an acute infusion of Veh, IGF-1, or IGF-1 and IGF-1+Let to the lateral ventricle. Either 1 or 24 h later, hippocampi were processed for subcellular fractionation and Western blotting for phosphorylated levels of pS118-ERα, total ERα, and nuclear loading control CREB in the nuclear fraction of all samples. Levels of pS118 were normalized to total ERα levels, and levels of total ERα were normalized to CREB levels. Graph represents mean D×A ± SEM expressed as a percentage of the vehicle group. ***A***, ***B***, There was no effect of treatment on pS118-ERα levels in the nuclear compartment either 1 h (***A***) or 24 h (***B***) after infusion. ***C***, There was no effect of treatment on pS118-ERα levels 24 h after infusion. ***D***, There was an effect of treatment (*p* < 0.05) on total ERα levels 24 h after infusion, with *post hoc* testing revealing increased levels in the IGF-1 group compared with the Veh group. *p < 0.05 versus Veh.

In the nuclear compartment (Fig. 4), there were no effects of treatment on levels of pS118-ERα (Fig. 4*A*; *F*_(2,27)_ = 0.095, *p* = 0.910) or total ERα (Fig. 4*B*; *F*_(2,27)_ = 0.202, *p* = 0.818) 1 h after infusion. At the 24 h time point, there was no effect of treatment on pS118-ERα levels in the nuclear compartment (Fig. 4*C*; *F*_(2,27)_ = 0.084, *p* = 0.919). However, there was an effect of treatment on total ERα levels in the nuclear compartment (Fig. 4*D*; *F*_(2,27)_ = 3.915, *p* = 0.033) 24 h after infusion, with the IGF-1 treatment group showing significantly higher levels compared with the vehicle group (*p* = 0.011) and near significantly higher levels compared with the IGF-1+Let group (*p* = 0.079). There was no significant difference in total ERα levels between the vehicle and IGF-1+Let treatment groups (*p* = 0.388).

In summary, results reveal that IGF-1-mediated phosphorylation of cytosolic ERα and subsequent increase in total levels of nuclear ERα require neuroestrogen synthesis.

#### MAPK and Akt signaling

To determine whether IGF-1 activation of ERα occurs via the MAPK or PI3K-Akt signaling pathways and whether IGF-1 activation of pathways requires neuroestrogen synthesis, we measured phosphorylated and total levels of p44-MAPK (ERK-1), p42-MAPK (ERK-2), and Akt.

**Figure 5.**
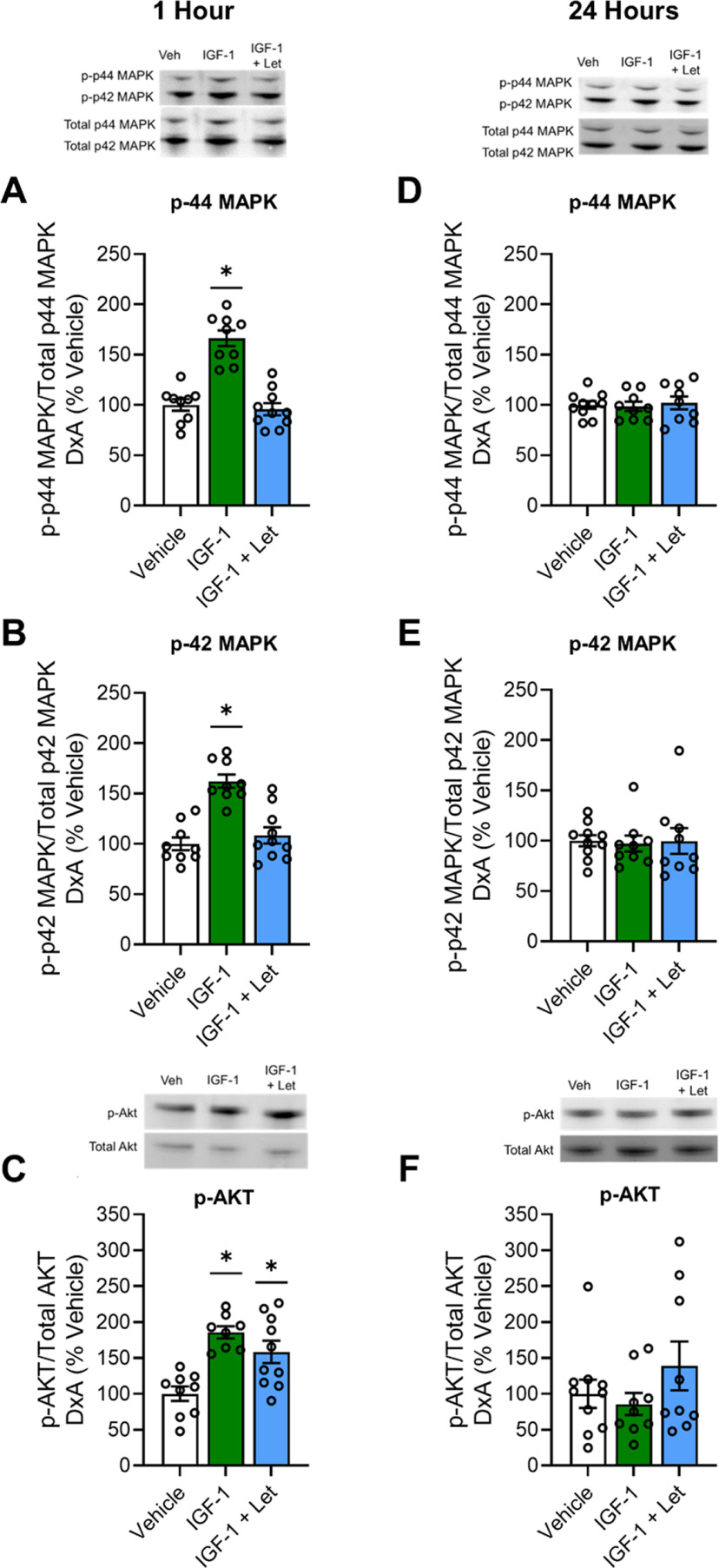
Hippocampal MAPK and Akt pathway activation 1 or 24 h after infusion of IGF-1 or IGF-1+Let. Middle-aged female rats were ovariectomized and given an acute infusion of Veh, IGF-1, or IGF-1 and IGF-1+Let to the lateral ventricle. Either 1 or 24 h later, hippocampi were processed for Western blotting for phosphorylated and total levels of p44-MAPK, p42-MAPK, and Akt. Phosphorylated levels were normalized to the total protein levels. Graph represents mean D×A ± SEM expressed as a percentage of the vehicle group. ***A–D***, One hour after infusion, there was a main effect of treatment (*p* < 0.05) on phosphorylated levels of p44-MAPK (***A***) and p42-MAPK (***B***), with *post hoc* testing revealing increased phosphorylation of both MAPK phosphorylation sites in the IGF-1 group compared with the Veh group. There was no effect of treatment on phosphorylated levels of p44-MAPK (***D***) or p42-MAPK (***E***) 24 h after infusion. ***C***, One hour after infusion, there was an effect (*p* < 0.05) of treatment on phosphorylated levels of Akt, with *post hoc* testing revealing increased levels in both the IGF-1 and IGF-1+Let groups compared with the Veh group. ***F***, There was no effect of treatment on phosphorylated levels of Akt 24 h after infusion. *p < 0.05 versus Veh.

One hour after infusion, there was a main effect of treatment on phosphorylation of both p44 (Fig. 5*A*; *F*_(2,27)_ = 35.750, *p* < 0.001) and p42 (Fig. 5*B*; *F*_(2,27)_ = 21.720, *p* < 0.001) phospho-sites of MAPK. *Post hoc* testing revealed a significant increase in phosphorylation of both phospho-sites of MAPK in the IGF-1 treatment group compared with the Vehicle group (p44-MAPK, *p* < 0.001; p42-MAPK, *p* < 0.001) and the IGF-1+Let group (p44-MAPK, *p* < 0.001; p42-MAPK, *p* < 0.001). There was no difference between the IGF-1+Let group and the Veh group on phosphorylation of either p44 (*p* = 0.647) or p42 (*p* = 0.407) MAPK levels.

One hour after infusion, there was a main effect of treatment on phosphorylation of Akt (Fig. 5*C*; *F*_(2,27)_ = 7.552, *p* = 0.003). *Post hoc* testing revealed a significant increase in phosphorylation of Akt in the IGF-1 group (*p* = 0.001) and the IGF-1+Let group (*p* = 0.006) compared with the Veh group.

Twenty-four hours after infusion, there was no main effect of treatment on phosphorylation of p44 MAPK (Fig. 5*D*; *F*_(2,27)_ = 0.120, *p* = 0.888), p42 MAPK (Fig. 5*E*; *F*_(2,27)_ = 0.031, *p* = 0.970), or Akt (Fig. 5*F*; *F*_(2,27)_ = 1.247, *p* = 0.297).

In summary, results reveal that, in parallel to impacts of IGF-1 on ERα activation, IGF-1-mediated activation of MAPK, but not Akt, requires neuroestrogen synthesis.

### Experiment 2: role of neuroestrogens in the ability of IGF-1 to impact spatial memory and associated signaling pathways

Experiment 1 revealed that acute IGF-1 activation of the MAPK signaling pathway, associated phosphorylation of ERα, and subsequent increase in levels of ERα in the hippocampus were blocked by letrozole, suggesting that they require neuroestrogen synthesis. In Experiment 2, we determined the functional consequences of these effects by testing the hypothesis that the ability of IGF-1 to impact hippocampal-dependent memory in recently ovariectomized rats also requires concomitant neuroestrogen synthesis. Additionally, we determined whether IGF-1 activation of signaling pathways in these behaviorally tested animals would parallel effects on memory. Following training on the radial maze, recently ovariectomized middle-aged rats received chronic intracerebroventricular treatment of vehicle (Veh group), IGF-1 (IGF-1 group), or IGF-1 plus letrozole (IGF-1+Let group) and were tested on delay trials in the maze. Following maze testing, hippocampal levels of MAPK and PI3K-Akt pathway activation were measured.

#### Spatial memory

**Figure 6.**
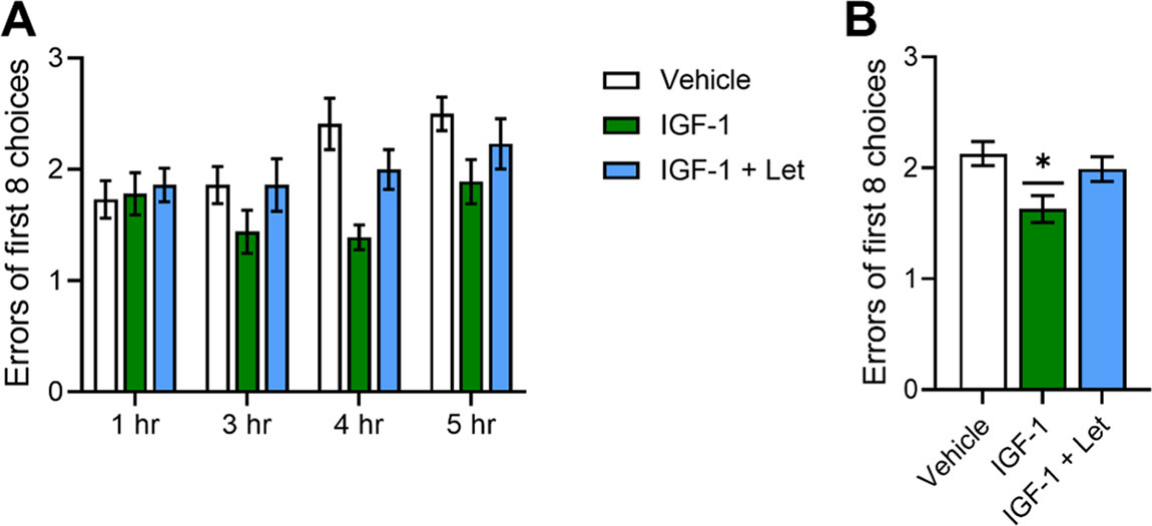
Impacts of chronic IGF-1 or IGF-1+Let treatment on performance on the hippocampal-dependent radial-arm maze task. Middle-aged female rats were trained on the 8-arm radial maze task and subsequently ovariectomized and treated with Veh, IGF-1, or IGF-1+Let and tested on the maze using delays of 1, 3, 4, and 5 h. Data represent the number of incorrect choices made in the first eight choices averaged across 2 d of testing at each delay. Graph represents mean errors of the first eight choices ± SEM. ***A***, There was a main effect of delay (*p* < 0.05) on performance across groups, with *post hoc* testing revealing errors increased as delays became longer. There was no significant interaction between delay × treatment. ***B***, There was a main effect of treatment (*p* < 0.05) on performance averaged across all delays, with *post hoc* testing revealing significantly fewer errors in the IGF-1 group compared with the Veh group. *p < 0.05 versus Veh.

Following recovery from stereotaxic surgeries, animals were tested across multiple increasing delays (1, 3, 4, 5 h) on the 8-arm radial maze test. As illustrated in Figure 6, mixed-design ANOVA revealed a main effect of delay (*F*_(3,84)_ = 4.257; *p* = 0.008) and a main effect of treatment (*F*_(2,28)_ = 5.245; *p* = 0.012) on radial-arm maze performance. *Post hoc* testing revealed significantly fewer errors of 8 across delays in the IGF-1 group compared with both the Veh group (*p* = 0.004) and the IGF-1+Let group (*p* = 0.029). There was no difference between the Veh group and the IGF-1+Let group. There was no significant interaction between delay and treatment (*F*_(6,84)_ = 1.555; *p* = 0.171). Thus, results reveal that IGF-1 administration in recently ovariectomized rats enhances performance on the radial-arm maze and that enhancement requires neuroestrogen synthesis.

#### MAPK and Akt signaling

**Figure 7.**
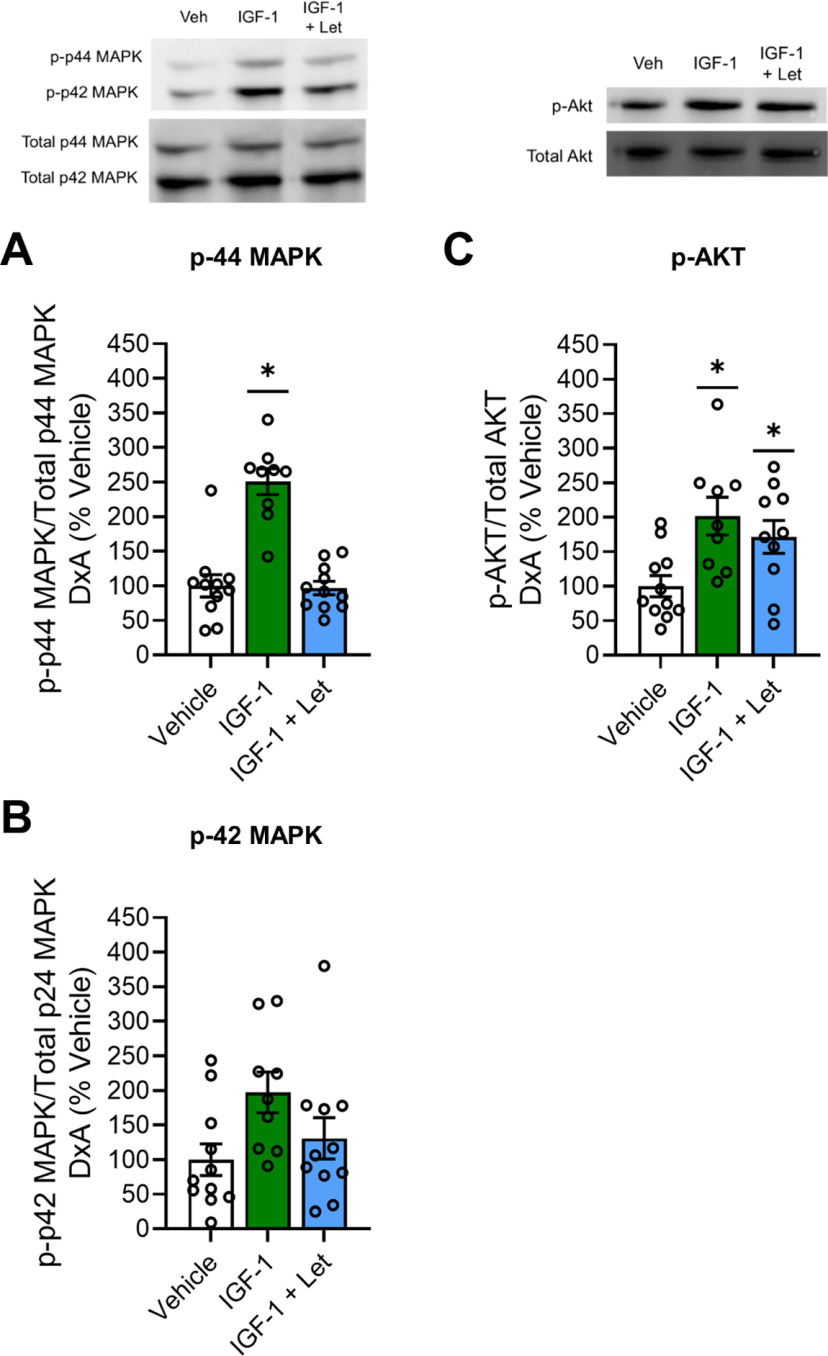
Impacts of chronic IGF-1 or IGF-1+Let treatment on hippocampal MAPK and Akt pathway activation. Middle-aged female rats were ovariectomized and treated with Veh, IGF-1, or IGF-1+Let. After rats were tested on the radial-arm maze, hippocampi were dissected and processed for Western blotting for phosphorylated and total levels of p44-MAPK, p42-MAPK, and Akt. Phosphorylated levels were normalized to the total protein levels and expressed as a percentage of the Veh group mean. Graph represents mean D×A ± SEM expressed as a percentage of the vehicle group. There was an effect of treatment (*p* < 0.05) on phosphorylated levels of p44-MAPK (***A***) and p42-MAPK (***B***), with *post hoc* testing revealing increased phosphorylation of both MAPK phosphorylation sites in the IGF-1 group compared with the Veh group. There was an effect of treatment (*p* < 0.05) on phosphorylated levels of Akt (***C***), with *post hoc* testing revealing increased phosphorylation in both the IGF-1 and IGF-1+Let groups compared with Veh group. *p < 0.05 versus Veh.

Following chronic intracerebroventricular treatment with either IGF-1 or IGF-1+Let, there was a main effect of treatment on phosphorylation of p44-MAPK (Fig. 7*A*; *F*_(2,30)_ = 32.346, *p* < 0.001) and a near significant effect of treatment on phosphorylation of p42-MAPK (Fig. 7*B*; *F*_(2,30)_ = 3.106, *p* = 0.060). *Post hoc* testing revealed a significant increase in phosphorylation of p44-MAPK levels in the IGF-1 group compared with both the Veh group (*p* < 0.001) and the IGF-1+Let group (*p* < 0.001). There was no difference between the IGF-1+Let group and the Veh group on phosphorylation of p44 (*p* = 0.878) MAPK levels.

As illustrated in Figure 7*C*, there was a main effect of treatment on phosphorylation of Akt (*F*_(2,30)_ = 4.803, *p* = 0.016). *Post hoc* testing revealed a significant increase in phosphorylation of Akt in the IGF-1 group (*p* = 0.012) and the IGF-1+Let group (*p* = 0.013) compared with the Veh group. There was no difference in levels of phosphorylation of Akt between the IGF-1 and IGF-1+Let groups (*p* = 0.907).

In summary, results reveal that IGF-1 activation of MAPK, but not Akt, signaling requires neuroestrogen synthesis in recently ovariectomized, behaviorally tested rats.

### Experiment 3: independent and interactive effects of neuroestrogens and IGF-1 signaling on hippocampal function in aging females following long-term ovarian hormone deprivation

Experiments 1 and 2 revealed that IGF-1 enhancement of hippocampal-dependent memory and elevation of phosphorylated and total hippocampal levels of ERα in recently ovariectomized rats require concomitant neuroestrogen synthesis. Furthermore, results suggest that these effects are mediated via activation of the MAPK and not the PI3K-Akt signaling pathway. Previous evidence indicates that neuroestrogen synthesis is regulated by circulating estrogens (Nelson et al., 2016) and therefore, not surprisingly, long-term ovariectomy results in decreased aromatase expression and neuroestrogen levels (Ma et al., 2020; Chen et al., 2021). In Experiment 3, we aimed to determine the implications of decreased level of neuroestrogens resulting from long-term ovariectomy on IGF-1 signaling effects in the hippocampus. We used a rat model of menopause in which no postovariectomy estradiol was administered, modeling individuals who do not use menopausal hormone therapy. Because we aimed to assess effects of long-term loss of ovarian function on endogenous IGF-1 signaling and subsequent impact for cognitive aging, we chose to antagonize IGF-1 here rather than exogenously administer IGF-1, as was done in Experiments 1 and 2. Long-term ovariectomized rats (100 d) that received no estradiol treatment received chronic intracerebroventricular delivery of vehicle, the IGF-1R antagonist JB1, aromatase inhibitor letrozole, or JB1+Let and were tested on the radial-maze task. Hippocampal levels of MAPK and PI3K-Akt pathway activation were measured. Finally, hippocampal expression of ERα, aromatase (the enzyme that converts testosterone to estradiol) and estradiol levels were measured.

#### Spatial memory

**Figure 8.**
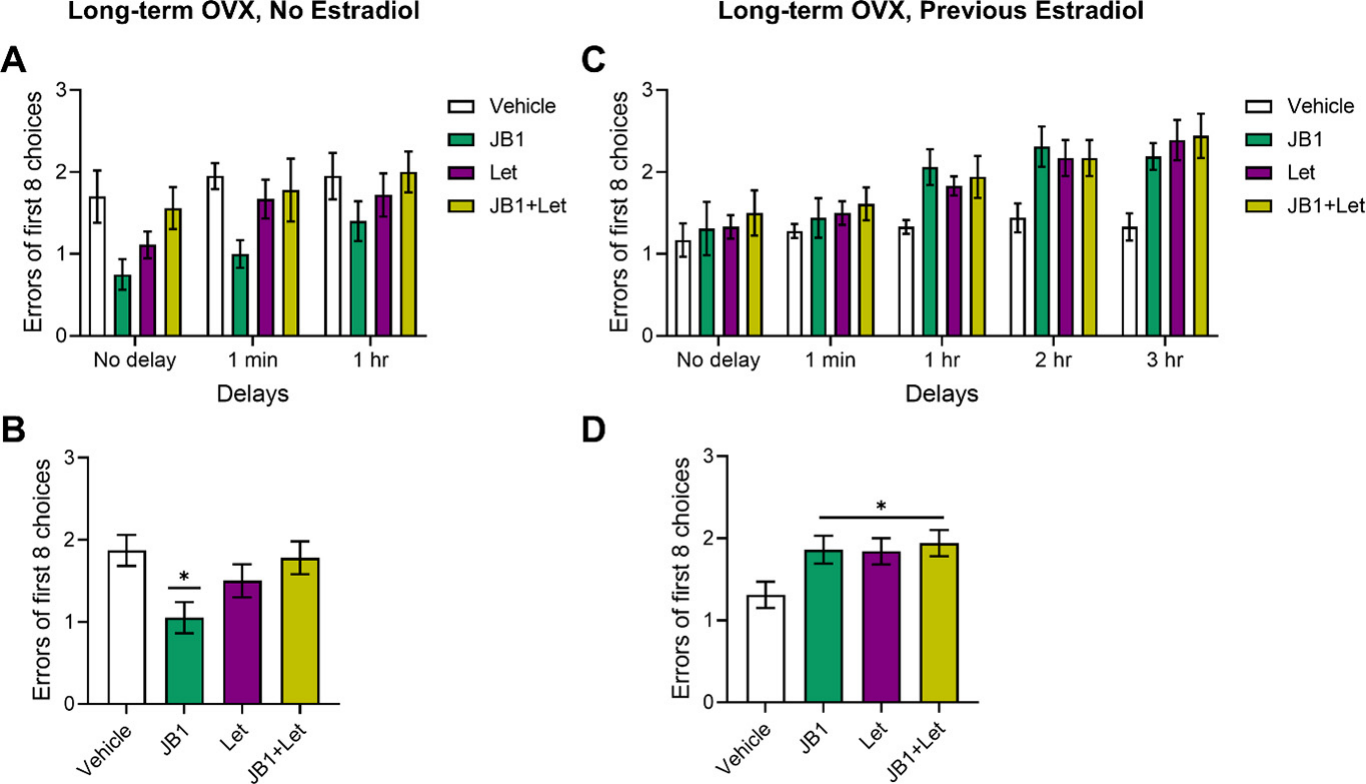
Impacts of chronic JB1, letrozole, or JB1+Let treatment on radial-arm maze performance in long-term ovariectomized rats with or without previous midlife estradiol exposure. Middle-aged female rats were ovariectomized and immediately implanted with vehicle (Long-term OVX, No Estradiol) or estradiol (Long-term OVX, Previous Estradiol) capsules. Forty days later, capsules were removed. One hundred days following loss of circulating estrogens (via ovariectomy in Long-term OVX, No Estradiol group or removal of estradiol capsule in Long-term OVX, Previous Estradiol group), rats were trained on the 8-arm radial maze task. Following training, rats were treated with chronic intracerebroventricular administration of Veh, the IGF-1R antagonist JB1, the aromatase inhibitor letrozole (Let), or JB1 and letrozole (JB1+Let) and tested on the maze using delays (Long-term OVX, No Estradiol: No Delay, 1 min, and 1 h; Long-term OVX, Previous Estradiol: No Delay, 1 min, 1, 2, and 3 h). Data represent the number of incorrect choices made in the first eight choices averaged across 2 d of testing at each delay. Graph represents mean errors of the first eight choices ± SEM. ***A***, Following long-term ovariectomy with no estradiol exposure, there was a main effect of delay (*p* < 0.05) on performance across groups, with *post hoc* testing revealing errors increased as delays became longer. There was no significant interaction between delay × treatment. ***B***, There was a main effect of treatment (*p* < 0.05) on performance averaged across all delays following long-term ovariectomy with no estradiol, with *post hoc* testing revealing significantly fewer errors in the JB1 group compared with the Veh group. ***C***, Following long-term ovariectomy with previous estradiol exposure, there was a main effect of delay (*p* < 0.05) on performance across groups, with *post hoc* testing revealing errors increased as delays became longer. There was no significant interaction between delay × treatment. ***D***, There was a main effect of treatment (*p* < 0.05) on performance averaged across all delays following previous estradiol exposure, with *post hoc* testing revealing significantly more errors in the JB1, Let, and JB1+Let groups compared with the Veh group. *p < 0.05 versus Veh.

As illustrated in Figure 8, mixed-design ANOVA revealed a main effect of delay (*F*_(2,68)_ = 6.713; *p* = 0.002) and a main effect of treatment (*F*_(3,34)_ = 3.702; *p* = 0.021) on radial-arm maze performance. *Post hoc* testing revealed significantly fewer errors of 8 across delays in the JB1 group compared with the Veh group (*p* = 0.012). There was no difference between the Veh group and the letrozole (*p* = 0.417) or between the Veh group and the JB1+Let group (*p* = 0.978). There was no significant interaction between delay and treatment (*F*_(6,68)_ = 0.419; *p* = 0.864). Results reveal that antagonizing IGF-1 receptor activity unexpectedly enhances spatial memory, suggesting that long-term ovariectomy leads to negative impacts of IGF-1 signaling on memory. Inhibition of neuroestrogens reverses the enhancement but has no impact on its own.

#### MAPK and Akt signaling

**Figure 9.**
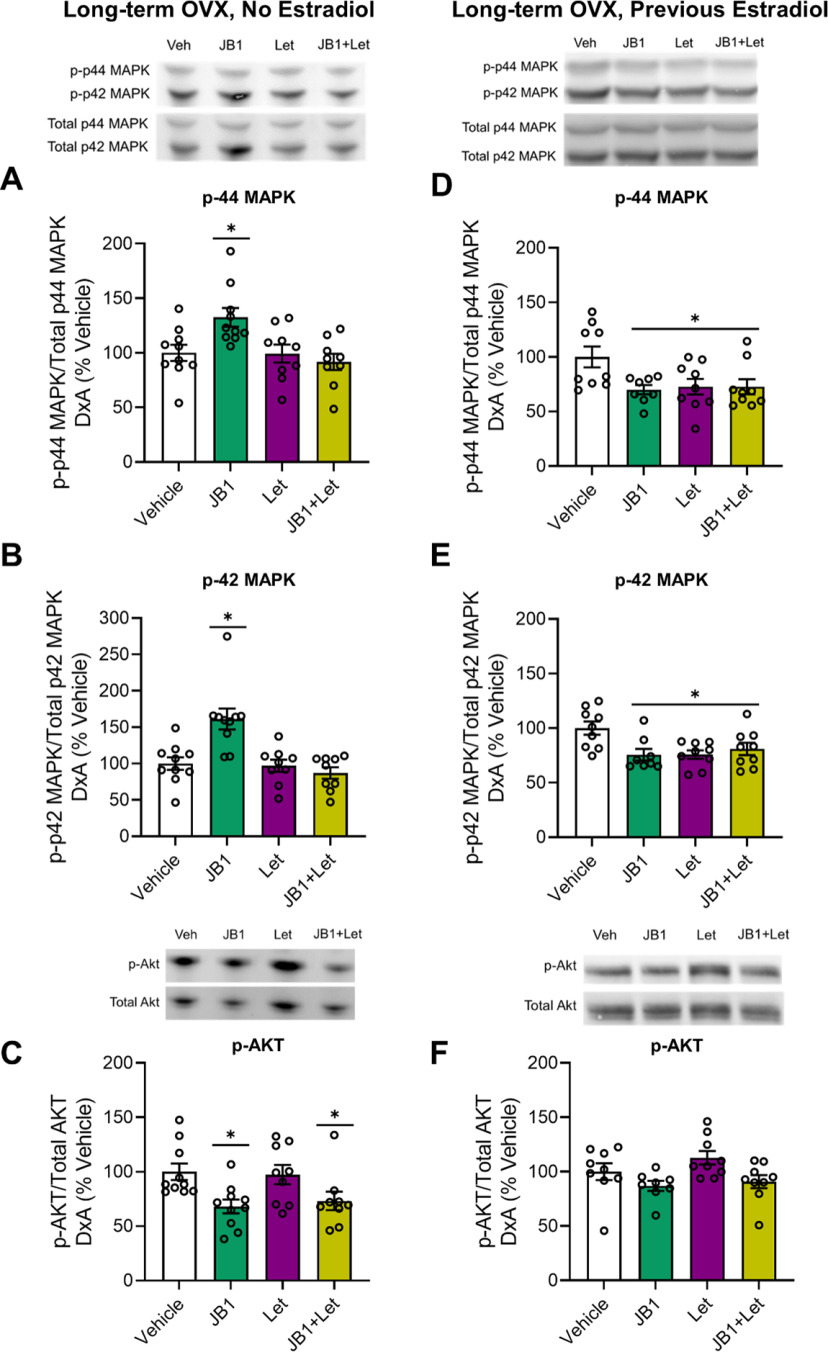
Impacts of chronic JB1, letrozole, or JB1+Let treatment on hippocampal MAPK and Akt pathway activation in long-term ovariectomized rats with or without previous midlife estradiol exposure. Middle-aged female rats were ovariectomized and immediately implanted with vehicle (Long-term OVX, No Estradiol groups) or estradiol (Long-term OVX, Previous Estradiol groups) capsules. Forty days later, capsules were removed. One hundred days following loss of circulating estrogens (either via ovariectomy in Long-term OVX, No Estradiol group or removal of estradiol capsule in Long-term OVX, Previous Estradiol group), rats were trained on the 8-arm radial maze task and subsequently treated with chronic intracerebroventricular administration of Veh, the IGF-1R antagonist JB1 (JB1), the aromatase inhibitor letrozole (Let), or JB1 and letrozole (JB1+Let). After rats were tested on the radial-arm maze, hippocampi were dissected and processed for Western blotting for phosphorylated and total levels of p44-MAPK, p42-MAPK, and Akt. Phosphorylated levels were normalized to the total protein levels. Graph represents mean D×A ± SEM expressed as a percentage of the vehicle group. Following long-term ovariectomy with no estradiol exposure, there was an effect of treatment (*p* < 0.05) on phosphorylated levels of p44-MAPK (***A***) and p42-MAPK (***B***), with *post hoc* testing revealing increased phosphorylation of both MAPK phosphorylation sites in the JB1 group compared with the Veh group. There was also an effect of treatment (*p* < 0.05) on phosphorylated levels of Akt (***C***), with *post hoc* testing revealing decreased phosphorylation in both the JB1 and JB1+Let groups compared with Veh group. Following long-term ovariectomy with previous estradiol exposure, there was an effect of treatment (*p* < 0.05) on phosphorylated levels of p44-MAPK (***D***) and p42-MAPK (***E***), with *post hoc* testing revealing decreased phosphorylation of both MAPK phosphorylation sites in the JB1, Let, and JB1+Let groups compared with the Veh group. There was also an effect of treatment (*p* < 0.05) on phosphorylated levels of Akt (***F***), with *post hoc* testing revealing no significant difference between the treatment groups and the Veh group. *p < 0.05 versus Veh.

After chronic treatment with JB1, letrozole, or JB1+Let following long-term ovariectomy, there was an effect of treatment on phosphorylation of both p44-MAPK (Fig. 9*A*; *F*_(3,37)_ = 5.367, *p* = 0.004) and p42-MAPK (Fig. 9*B*; *F*_(3,37)_ = 10.793, *p* < 0.001). *Post hoc* testing revealed a significant increase in phosphorylation of p44-MAPK (*p* = 0.015) and p42-MAPK (*p* < 0.001) levels in the JB1 group compared with the Veh group. There were no differences between the Veh group and the letrozole group for either p44-MAPK (*p* = 1.00) or p42-MAPK (*p* = 0.995), nor were there any differences between the Veh group and the JB1+Let group for either p44-MAPK (*p* = 0.805) or p42-MAPK (*p* = 0.709).

As illustrated in Figure 9*C*, there was an effect of treatment on phosphorylation of Akt (*F*_(3,37)_ = 4.437, *p* = 0.010). *Post hoc* testing revealed a significant decrease in phosphorylation of Akt in the JB1 group (*p* = 0.015) and a near significant decrease in phosphorylation of Akt in the JB1+Let group (*p* = 0.056) compared with the Veh group. There was no difference in levels of phosphorylation of Akt between the Veh group and the letrozole group (*p* = 0.990).

In summary, results reveal that antagonizing IGF-1 receptor activity increases MAPK signaling and decreases Akt signaling, suggesting that, under conditions of long-term ovarian hormone deprivation, PI3K-Akt signaling pathway predominates. Inhibition of neuroestrogen synthesis reverses JB1-induced effects on MAPK, but not on Akt.

#### Protein levels of ERα and aromatase

**Figure 10.**
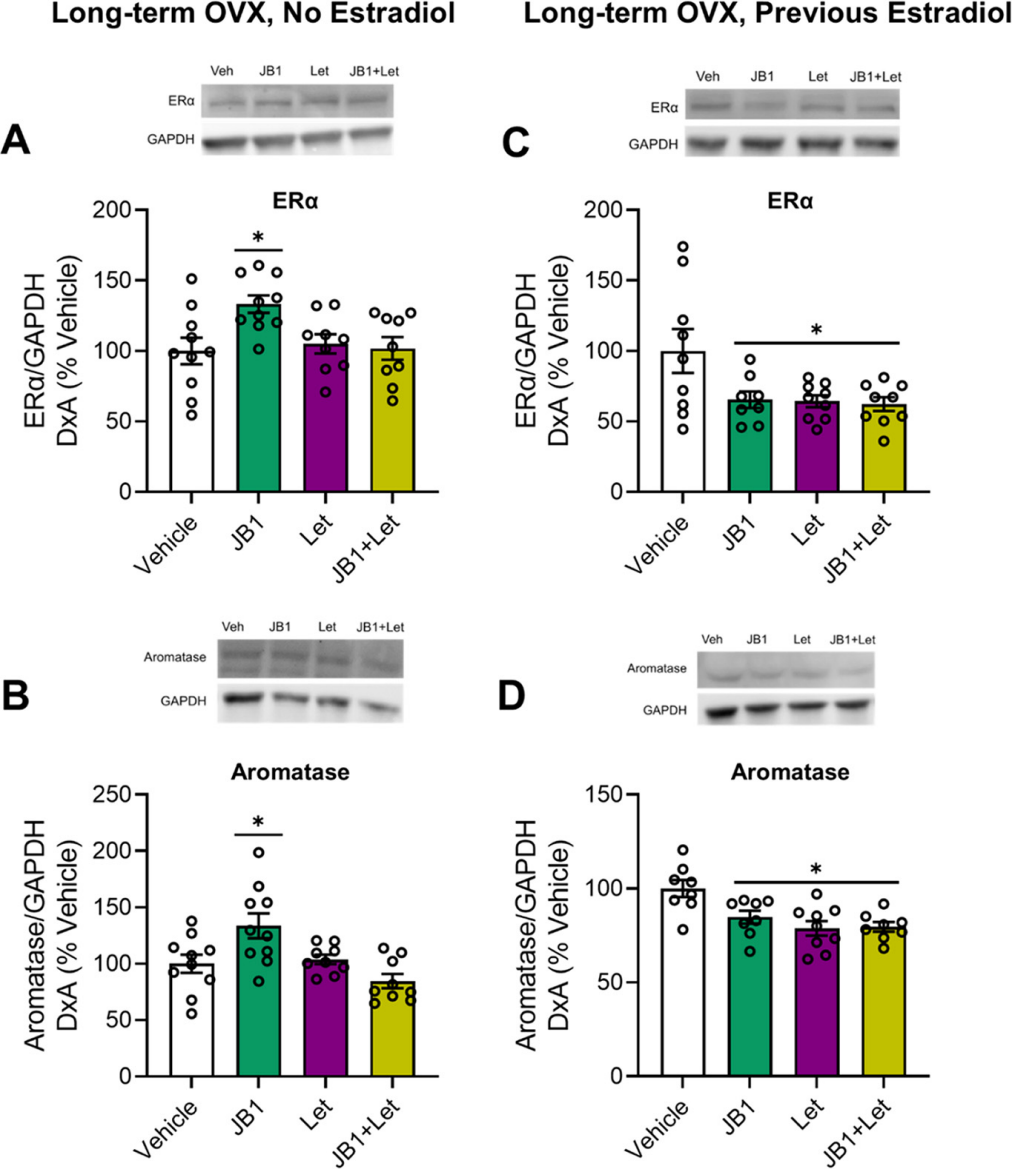
Impacts of chronic JB1, letrozole, or JB1+Let treatment on hippocampal ERα and aromatase levels in long-term ovariectomized rats with or without previous midlife estradiol exposure. Middle-aged female rats were ovariectomized and immediately implanted with vehicle (Long-term OVX, No Estradiol groups) or estradiol (Long-term OVX, Previous Estradiol groups) capsules. Forty days later, capsules were removed. One hundred days following loss of circulating estrogens (either via ovariectomy in Long-term OVX, No Estradiol group or removal of estradiol capsule in Long-term OVX, Previous Estradiol group), rats were trained on the 8-arm radial maze task and subsequently treated with chronic intracerebroventricular administration of Veh, the IGF-1R antagonist JB1 (JB1), the aromatase inhibitor letrozole (Let), or JB1+Let. After rats were tested on the radial-arm maze, hippocampi were dissected and processed for Western blotting for ERα, aromatase, and loading control GAPDH. ERα and aromatase levels were normalized to GAPDH levels. Graph represents mean D×A ± SEM expressed as a percentage of the vehicle group. Following long-term ovariectomy with no estradiol exposure, there was an effect of treatment (*p* < 0.05) on hippocampal ERα (***A***) and aromatase (***B***) expression, with *post hoc* testing revealing increased levels of both proteins in the JB1 group compared with the Veh group. Following long-term ovariectomy with previous estradiol exposure, there was an effect of treatment (*p* < 0.05) on hippocampal ERα (***C***) and aromatase (***D***) expression with *post hoc* testing revealing decreased levels in the JB1, Let, and JB1+Let groups compared with the Veh group. *p < 0.05 versus Veh.

As illustrated in Figure 10*A*, there was an effect of treatment on hippocampal ERα levels (*F*_(3,37)_ = 4.202, *p* = 0.012). *Post hoc* testing revealed a significant increase in ERα expression in the JB1 group (*p* = 0.011) compared with the Veh group. There was no difference in ERα levels between the Veh group and the letrozole group (*p* = 0.940) or between the Veh group and the JB1+Let group (*p* = 0.997).

There was a main effect of treatment on hippocampal aromatase levels, as shown in Figure 10*B* (*F*_(3,37)_ = 6.65, *p* = 0.001). *Post hoc* testing revealed a significant increase in aromatase expression in the JB1 group (*p* = 0.012) compared with the Veh group. There was no difference in aromatase levels between the Veh group and the letrozole group (*p* = 0.974) or between the Veh group and the JB1+Let group (*p* = 0.403).

In summary, results reveal that antagonism of IGF-1 receptors increases protein levels of ERα and aromatase in the hippocampus, effects reversed by inhibition of neuroestrogen synthesis.

#### Hippocampal estradiol levels

**Figure 11.**
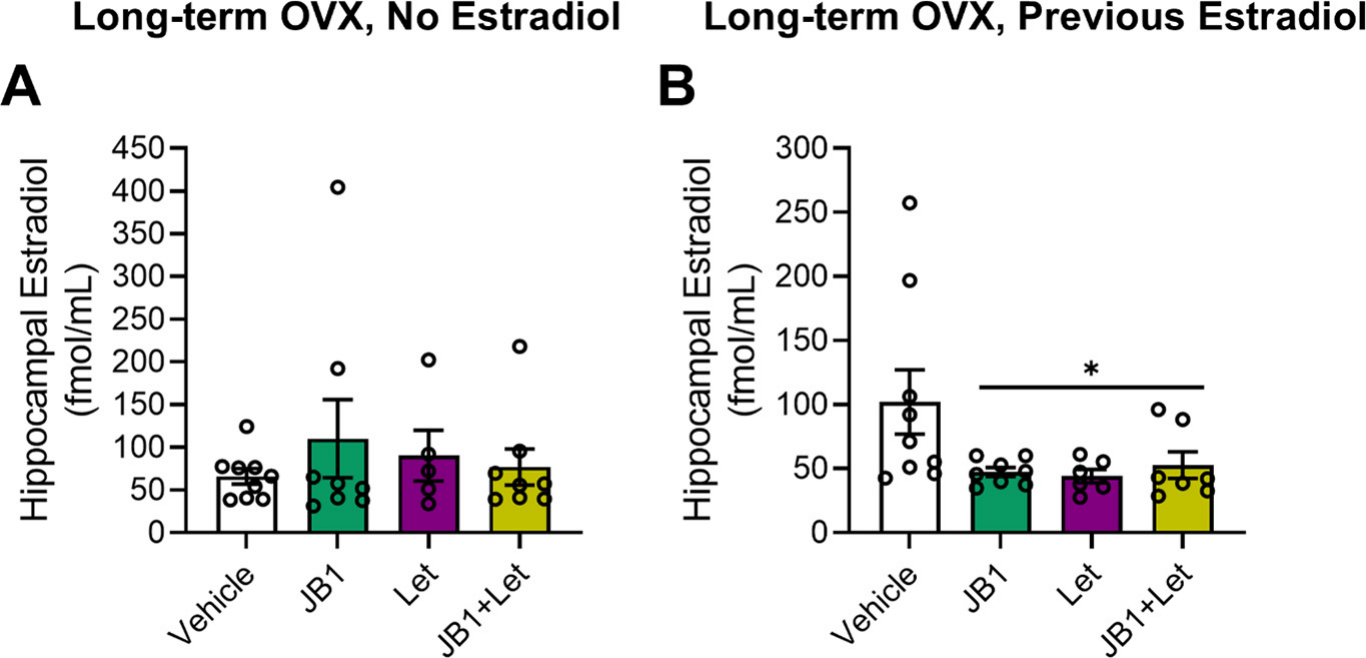
Impacts of chronic JB1, letrozole, or JB1+Let treatment on hippocampal estradiol levels in long-term ovariectomized rats with or without previous midlife estradiol exposure. Middle-aged female rats were ovariectomized and immediately implanted with vehicle (Long-term OVX, No Estradiol groups) or estradiol (Long-term OVX, Previous Estradiol groups) capsules. Forty days later, capsules were removed. One hundred days following loss of circulating estrogens (via ovariectomy in Long-term OVX, No Estradiol group or removal of estradiol capsule in Long-term OVX, Previous Estradiol group), rats were trained on the 8-arm radial maze task and subsequently treated with chronic intracerebroventricular administration of Veh, the IGF-1R antagonist JB1, the aromatase inhibitor letrozole (Let), or JB1 and letrozole (JB1+Let). After rats were tested on the radial-arm maze, hippocampi were dissected and processed for estradiol detection via UPLC-MS/MS. Estradiol levels are expressed in fmol/ml. Graph represents group means ± SEM expressed as a percentage of the vehicle group. ***A***, Following long-term ovariectomy with no estradiol exposure, there was no effect of treatment on hippocampal estradiol levels. ***B***, Following long-term ovariectomy with previous estradiol exposure, there as an effect of treatment (*p* < 0.05) on estradiol levels, with *post hoc* testing revealed decreased levels in the JB1, Let, and JB1+Let groups compared with the Veh group. *p < 0.05 versus Veh.

After chronic treatment with JB1, letrozole, or JB1+Let following long-term ovariectomy, there was no effect of treatment on hippocampal estradiol levels (Fig. 11*A*; *F*_(3,29)_ = 0.466, *p* = 0.708). Results reveal that neither antagonism of IGF-1 receptors nor inhibition of neuroestrogen synthesis impacted levels of locally synthesized neuroestrogens.

### Experiment 4: independent and interactive effects of neuroestrogens and IGF-1 signaling on hippocampal function in aging females with a history of previous estradiol administration

Results of Experiment 3 revealed that long-term ovarian deprivation disrupts the ability of IGF-1 and neuroestrogens to exert positive interactive effects on memory as indicated by an enhancement resulting from IGF-1 receptor antagonism and a lack of disruptive effects on memory of letrozole treatment alone. In contrast to the Experiment 3 results in which JB1 enhanced memory, previous work from our laboratory revealed that antagonism of IGF-1 receptors by JB1 disrupts memory in ovariectomized rats treated with ongoing (Nelson et al., 2014) or previous (Witty et al., 2013) estradiol. In Experiment 4, we tested the hypothesis that previous midlife estradiol exposure maintains the positive impact of IGF-1 signaling on the MAPK signaling pathway, ERα levels, and subsequent impact on memory by sustaining neuroestrogen synthesis in the hippocampus long-term, even after termination of estradiol treatment and in the absence of circulating estrogens. We used a rat model of menopause in which middle-aged animals received an estradiol implant at the time of ovariectomy that was removed following 40 d of treatment, modeling individuals who take hormones for a few years and then stop. One hundred days following termination of estradiol treatment, rats were treated with chronic intracerebroventricular delivery of vehicle (Veh group), IGF-1R antagonist JB1, aromatase inhibitor letrozole, or JB1+Let. Rats were then tested on a hippocampal-dependent spatial memory task; and later, hippocampal levels of MAPK and PI3K-Akt pathway activation, ERα and aromatase expression, and estradiol levels were measured.

#### Spatial memory

As illustrated in Figure 8, mixed-design ANOVA revealed a main effect of delay (*F*_(4,124)_ = 20.720; *p* < 0.001) and a main effect of treatment (*F*_(3,31)_ = 3.205; *p* = 0.037) on radial-arm maze performance. *Post hoc* testing revealed significantly more errors of 8 across delays in the JB1 group (*p* = 0.033), the letrozole group (*p* = 0.033), and the JB1+Let group (*p* = 0.012) compared with the Veh group. There was a statistically trending interaction between delay and treatment (*F*_(12,124)_ = 1.655; *p* = 0.085). Thus, antagonizing IGF-1 receptor activity, inhibiting neuroestrogen synthesis, or the combination of both exerts similar detrimental effects on spatial memory. Results indicate that a previous history of estradiol treatment allows for long-term maintenance of the beneficial interactive effects of IGF-1 and neuroestrogens in the hippocampus, in which both are necessary, but neither sufficient, to enhance memory.

#### MAPK and Akt signaling

After chronic treatment with JB1, letrozole, or JB1+Let following previous estradiol exposure, there was an effect of treatment on phosphorylation of both p44-MAPK (Fig. 9*D*; *F*_(3,34)_ = 3.694, *p* = 0.022) and p42-MAPK (Fig. 9*E*; *F*_(3,34)_ = 4.839, *p* = 0.007). *Post hoc* testing revealed a significant decrease in phosphorylation of both phosphorylation sites of MAPK in the JB1 group (p44-MAPK, *p* = 0.022; p42-MAPK, *p* = 0.008), the letrozole group (p44-MAPK, *p* = 0.035; p42-MAPK, *p* = 0.007), and the JB1+Let group (p44-MAPK, *p* = 0.034; p42-MAPK, *p* = 0.038) compared with the Veh group.

As illustrated in Figure 9*F*, there was an effect of treatment on phosphorylation of Akt (*F*_(3,34)_ = 3.248, *p* = 0.035). However, *post hoc* testing revealed no significant differences between the Veh group and the JB1 (*p* = 0.363), letrozole (*p* = 0.350), or JB1+Let (*p* = 0.615) groups.

Results reveal that antagonizing IGF-1 receptor activity, inhibiting neuroestrogen synthesis, or the combination of both, resulted in similar decreased levels of MAPK activation, and no effects of on Akt activation. Thus, after a previous history of estradiol treatment, MAPK signaling pathway predominates because of interactions between IGF-1 and neuroestrogen signaling.

#### Protein levels of ERα and aromatase

As illustrated in Figure 10*C*, there was an effect of treatment on hippocampal ERα levels (*F*_(3,34)_ = 4.008, *p* = 0.016). *Post hoc* testing revealed a significant decrease in ERα expression in the JB1 group (*p* = 0.034), the letrozole group (*p* = 0.023), and the JB1+Let group (*p* = 0.016) compared with the Veh group.

Finally, there was an effect of treatment on hippocampal aromatase levels, as shown in Figure 10*D* (*F*_(3,34)_ = 8.803, *p* < 0.001). *Post hoc* testing revealed a significant decrease in aromatase expression in the JB1 group (*p* = 0.009), the letrozole group (*p* < 0.001), and the JB1+Let group (*p* < 0.001) compared with the Veh group.

Results reveal that antagonizing IGF-1 receptor activity, inhibiting neuroestrogen synthesis, or the combination of both lead to similar decreases in protein levels of ERα and aromatase in the hippocampus.

#### Hippocampal estradiol levels

After chronic treatment with JB1, letrozole, or JB1+Let following previous estradiol exposure, there was an effect of treatment on hippocampal estradiol levels (Fig. 11*B*; *F*_(3,29)_ = 3.10, *p* = 0.044). *Post hoc* testing revealed a significant decrease in estradiol expression in the JB1 group (*p* = 0.024), the letrozole group (*p* = 0.027), and the JB1+Let group (*p* = 0.048) compared with the Veh group. Thus, antagonizing IGF-1 receptor activity has similar effects on hippocampal neuroestrogen levels as direct inhibition of its synthesis. Results suggest that, following a period of previous midlife estradiol exposure, IGF-1 signaling helps maintain neuroestrogen levels.

## Discussion

Results reveal that short-term estrogen treatment during midlife, as is commonly used during the menopausal transition in humans, provides lasting benefits for hippocampal function and memory by robustly altering the interactive relationship between IGF-1 and locally synthesized neuroestrogens in mediating ligand-independent activation of hippocampal ERα. First, we showed in recently ovariectomized rats (∼10 d) that neuroestrogen synthesis is required for IGF-1-mediated increases in phosphorylation of ERα, activation of the MAPK pathway, and enhanced performance on the hippocampal-dependent radial-arm maze. Next, we found that, following long-term ovariectomy (∼100 d), IGF-1 signaling and neuroestrogen signaling no longer provided the same benefits for hippocampal function and memory, demonstrating a weakened relationship between the two hormones following long periods of ovarian hormone deprivation. Remarkably, short-term (40 d) treatment with estradiol immediately following ovariectomy successfully maintained the relationship between IGF-1 and neuroestrogen signaling, resulting in enhanced memory, increased hippocampal activation of MAPK, protein expression of ERα and aromatase, and estradiol levels. Together, results provide a potential model for combatting postmenopausal cognitive decline in which short-term estradiol treatment near the loss of ovarian hormones can sustain hippocampal function and memory by maintaining the dynamic relationships between ERα, IGF-1R, and neuroestrogen synthesis in the aging female brain.

### Effects of IGF-1 on ERα activation, MAPK signaling, and memory rely on local estrogen production

Results of Experiment 1 revealed that infusion of IGF-1 to brains of ovariectomized rats increased phosphorylation of hippocampal ERα at S118, a site associated with decreased degradation (Valley et al., 2005) and increased transcriptional activity (Dutertre and Smith, 2003) of the receptor. Subcellular compartment fractionation allowed us to localize the increased pS118-ERα that occurred 1 h following IGF-1 infusion to the cytosolic compartment of hippocampal cells. Results are consistent with *in vitro* work demonstrating peak dimerization (and presumably, therefore, nuclear translocation) of ERα does not occur until 2 h after estrogen treatment (Powell et al., 2010). Twenty-four hours after infusion of IGF-1, overall ERα levels were increased in the nuclear compartment of hippocampal cells. Results suggest that IGF-1 activation of ERα via phosphorylation at S118 promotes nuclear translocation of ERα, protecting the receptor from degradation and allowing for sustained ERα levels. Furthermore, results implicate a role for locally synthesized neuroestrogens in IGF-1 effects. Inhibition of local synthesis of neuroestrogens via administration of letrozole blocked the ability of IGF-1 to increase phosphorylation of ERα and the subsequent increase in nuclear ERα protein levels.

A potential mechanism by which IGF-1 and neuroestrogen interact to impact ERα is via intracellular signaling pathways. Both MAPK and PI3K-Akt signaling are activated via tyrosine kinase receptor IGF-1 or by neuroestrogens acting on membrane-bound estrogen receptors (Kadowaki et al., 1996; Boulware et al., 2005; Mendez and Garcia-Segura, 2006). Here, infusions of IGF-1, but not IGF-1 plus letrozole, increase phosphorylation of p44- and p42-MAPK. Letrozole had no impact on IGF-1-induced increase in Akt phosphorylation. Earlier work in cell culture demonstrated that MAPK phosphorylates ERα at S118 following IGF-1 treatment (Kato et al., 1995), and recent work from our laboratory in a neuroblastoma cell line supports the role of neuroestrogens in activating the MAPK pathway in conjunction with IGF-1R (Pollard and Daniel, 2019). Interestingly, Pollard and Daniel (2019) also demonstrated a mutually repressive relationship between MAPK and Akt in which both pathways inhibit each other, allowing for highly regulated control of ERα activity by IGF-1R. In summary, data indicate that IGF-1 and neuroestrogen signaling interact via the MAPK, but not the Akt, pathway, to activate hippocampal ERα in the absence of circulating estrogens. The significance of these interactions is supported by the results of Experiment 2 in which IGF-1-mediated enhancement of a hippocampal-dependent radial-maze task was blocked by letrozole, indicating that IGF-1 activation of ERα requires neuroestrogen synthesis to enhance hippocampal memory.

### Effects of IGF-1 on MAPK signaling and memory are significantly altered as a result of long-term loss of ovarian function and associated putative decrease in neuroestrogens

Local inhibition of aromatase activity has been shown to impair memory consolidation in recently ovariectomized mice (Tuscher et al., 2016). However, hippocampal aromatase expression (Ma et al., 2020), estradiol levels (Chen et al., 2021), and neuroestrogen-mediated transcriptional activity (Baumgartner et al., 2019) decrease following long-term, but not short-term, ovariectomy. Consistent with those findings are results of Experiment 3 in which blocking neuroestrogen synthesis via letrozole administration had no impact on hippocampal memory in long-term ovariectomized animals. Surprisingly, pharmacologically inhibiting IGF-1R using JB1 actually enhanced memory in long-term ovariectomized rats, suggesting the possibility that IGF-1 signaling becomes detrimental following long-term ovarian hormone deprivation and associated loss of local synthesis of neuroestrogens.

The paradoxical beneficial effect of IGF-1 antagonism on memory following long-term ovarian hormone deprivation could potentially be explained by regulation of aromatase expression via IGF-1 signaling. In addition to enhancement of memory, blocking IGF-1R with JB1 in long-term ovariectomized animals resulted in increased hippocampal MAPK activation, decreased PI3K-Akt activation, and increased ERα and aromatase levels. Importantly, JB1+Let did not have the same effects on memory and protein expression as JB1 administered alone, indicating that the positive impacts of JB1 on memory require subsequent neuroestrogen synthesis. While the precise mechanism for activation of the enzyme aromatase is far from clear, with different phospho-sites associated with increased enzymatic activity, stabilization of protein levels, or decreased enzymatic activity (Miller et al., 2008; Catalano et al., 2009; Charlier et al., 2011), its phosphorylation can be regulated by kinase cascades initiated by IGF-1R and membrane estrogen receptors. For example, in T47D breast cancer cells, inhibition of the Akt pathway was associated with increased aromatase activity (Su et al., 2011). Here, results suggest a mechanism in which inhibiting IGF-1R results in decreased PI3K-Akt activation, which in turn disinhibits the MAPK pathway and allows for increased ERα and aromatase stability. Ultimately, however, we detected no group differences in hippocampal estradiol levels following long-term ovariectomy, likely because of overall decreases in estradiol levels following long periods of ovarian hormone deprivation reported previously (Chen et al., 2021). Because the route of administration in the current studies was intracerebroventricular, there also remains a possibility that the baseline levels of hippocampal estradiol not impacted by drug treatments could be originating from peripheral sources, such as adipose or the adrenal glands (Barakat et al., 2016). Nevertheless, results suggest that a shift in IGF-1 signaling from MAPK to PI3K-Akt following long-term ovarian hormone deprivation is detrimental to memory.

### Effects of long-term loss of ovarian function are mitigated by early, short-term estrogen treatment, and reflect effects on levels of aromatase and neuroestrogens

Results of Experiment 4 demonstrate that a history of previous estradiol treatment reverses the negative effects of IGF-1 signaling on the hippocampus and memory in long-term ovariectomized rats. Consistent with earlier work (Witty et al., 2013), we found that JB1 treatment impaired memory and decreased levels of MAPK phosphorylation and ERα expression in animals previously treated with estradiol during midlife. Here we extend those findings by demonstrating the necessary role for neuroestrogens in facilitating activation of the MAPK pathway by IGF-1R. We found identical effects of JB1, letrozole, and JB1+Let on memory, MAPK phosphorylation, ERα and aromatase protein expression, and hippocampal estradiol levels in animals that experienced previous estradiol treatment, indicating that IGF-1R and neuroestrogens work together to maintain hippocampal function in aging females following a previous period of midlife estradiol treatment.

Although the current experiment designs prevent direct comparisons between Experiments 3 and 4, the differential effects of pharmacological manipulations between hormone treatments suggest diverging paths for hippocampal function in two models of menopause. On one path, long-term loss of ovarian hormones results in decreased neuroestrogen activity (Baumgartner et al., 2019), shifting the balance of IGF-1 signaling such that activation of the Akt pathway predominates over activation of the MAPK pathway, and leading to decreases in levels of aromatase and phosphorylation of ERα. On the other path, a short-term period of estradiol treatment immediately following loss of ovarian function reverses the negative impact of long-term hormone deprivation on hippocampal function (Rodgers et al., 2010; Witty et al., 2013; Baumgartner et al., 2021), potentially by sustaining levels of neuroestrogens well beyond the period of estradiol treatment, allowing for IGF-1-mediated activation of the MAPK pathway to predominate over the Akt pathway. In this model, MAPK signaling may contribute to sustained aromatase expression, continued neuroestrogen synthesis, and phosphorylation of ERα at phospho-site Ser-118. This activation via ligand-independent mechanisms results in dimerization and nuclear translocation of ERα, allowing for sustained levels of the receptor and leading to transcriptional changes that impact hippocampal function and ultimately enhance memory, all of which last far beyond the initial exposure to estradiol.

In conclusion, collectively, results indicate that short-term estrogen treatment following midlife loss of ovarian function has long-lasting effects on hippocampal function and memory by dynamically regulating cellular mechanisms that promote activity of ERα in the absence of circulating estrogens. Findings demonstrate how changes in hippocampal ERα expression, IGF-1R signaling, and neuroestrogen synthesis following long-term ovariectomy can negatively impact memory, but that a history of previous estradiol treatment protects the hippocampus against these changes to combat cognitive decline in rodent models of menopause.
